# Experimental and theoretical studies on structural changes in the microtubule affinity-regulating kinase 4 (MARK4) protein induced by *N*-hetarenes: a new class of therapeutic candidates for Alzheimer’s disease

**DOI:** 10.3389/fmed.2025.1529845

**Published:** 2025-03-19

**Authors:** Ashanul Haque, Khalaf M. Alenezi, Mohd. Saeed Maulana Abdul Rasheed, Md. Ataur Rahman, Saleha Anwar, Shahzaib Ahamad, Dinesh Gupta

**Affiliations:** ^1^Department of Chemistry, College of Science, University of Hail, Hail, Saudi Arabia; ^2^Department of Biology, College of Science, University of Hail, Hail, Saudi Arabia; ^3^Chemistry Program, New York University Abu Dhabi (NYUAD), Saadiyat Island, Abu Dhabi, United Arab Emirates; ^4^Centre for Interdisciplinary Research in Basic Sciences, Jamia Millia Islamia, New Delhi, India; ^5^Translational Bioinformatics Group, International Centre for Genetic Engineering and Biotechnology (ICGEB), New Delhi, India

**Keywords:** Alzheimer’s disease, Suzuki-Miyaura coupling, kinase inhibitor, pyrimidine derivatives, molecular docking, molecular dynamics

## Abstract

**Introduction:**

Alzheimer’s disease (AD) is a neurodegenerative disorder that progressively affects the cognitive function and memory of the affected person. Unfortunately, only a handful of effective prevention or treatment options are available today. Microtubule affinity-regulating kinase 4 (MARK4) is a serine/threonine protein that plays a critical role in regulating microtubule dynamics and facilitating cell division. The dysregulated expression of MARK4 has been associated with a range of diseases, including AD.

**Methods:**

In this study, we synthesized a series of *N*-hetarenes via Pd(0)-catalyzed Suzuki-Miyaura cross coupling reaction. All compounds were characterized using multi-spectroscopic techniques and evaluated for their activity against the MARK4 enzyme through ATPase inhibition assays. The experimental data was further supported by computational and quantum chemical calculations. We also computed the drug-likeness, bioavailability, and toxicity (ADME/T) profiles of the compounds.

**Results:**

Six new 4-(6-(arylpyrimidin-4-yl)piperazine-1-carboximidamides **5−10** were prepared in good yields. ATPase inhibition assay conducted on these compounds demonstrated IC_50_ values in micromolar range (5.35 ± 0.22 to 16.53 ± 1.71 μM). Among the tested compounds, 4-(6-(*p*-tolyl)pyrimidin-4-yl)piperazine-1-carboximidamide (**5**; IC_50_ = 5.35 ± 0.22 μM) and 4-(6-(benzo[*b*]thiophen-2-yl)pyrimidin-4-yl)piperazine-1-carboximidamide (**9**; IC_50_ = 6.68 ± 0.80 μM) showed the best activity. The binding constant (*K*), as determined by the fluorescence quenching assay was estimated to be 1.5 ± 0.51 × 10^5^ M^−1^ for **5** and 1.14 ± 0.26 × 10^5^ M^−1^ for **9**. The results of molecular docking and MD simulation studies against MARK4 (PDB: 5ES1) indicated that compounds were able to bind the ATP binding pocket of the MARK4, leading to its stabilization. Additionally, ADME/T analysis revealed a high degree of drug-likeness of the compounds.

**Conclusion:**

We demonstrated that 4-(6-(arylpyrimidin-4-yl)piperazine-1-carboximidamides) are a promising class of *N*-hetarenes for developing next-generation anti-AD drugs. The reported class of compounds inhibited MARK4 activity in-vitro at micromolar concentration by targeting the ATP-binding pocket. These findings provide valuable insights for future drug design.

## 1 Introduction

Alzheimer’s disease (AD) stands as the most prevalent neurodegenerative disorder linked to dementia, impacting millions of individuals globally (1). According to the reports from the Ministry of Health in Saudi Arabia, there are currently around 130,000 individuals living with AD. The number is projected to increase dramatically, with estimates suggesting it could triple over the next 30 years (2). This disease affects not only the day-to-day lifestyle of those affected but also has financial and psychological impacts on their families and caregivers, highlighting the urgent need for continued research and support in managing this complex condition (3). Decades of research in this area have identified aetiology implicated in AD progression (4). Drawing from this body of knowledge, researchers have identified various drugs that target one or more underlying pathways associated with this disease (5). However, many of these treatments are only effective during particular stages of the disease and can come with side effects (6). This has prompted scientists to pursue the discovery of new drug candidates with enhanced potency and selectivity. In pursuit of this goal, researchers have extensively examined various synthetic (7) and natural (8) compounds that engage different signaling pathways related to AD. Although there have been several promising findings in recent studies, the overall clinical effectiveness and outcomes of these new agents remain uncertain, stressing the need for further investigation.

**Figure 1:**
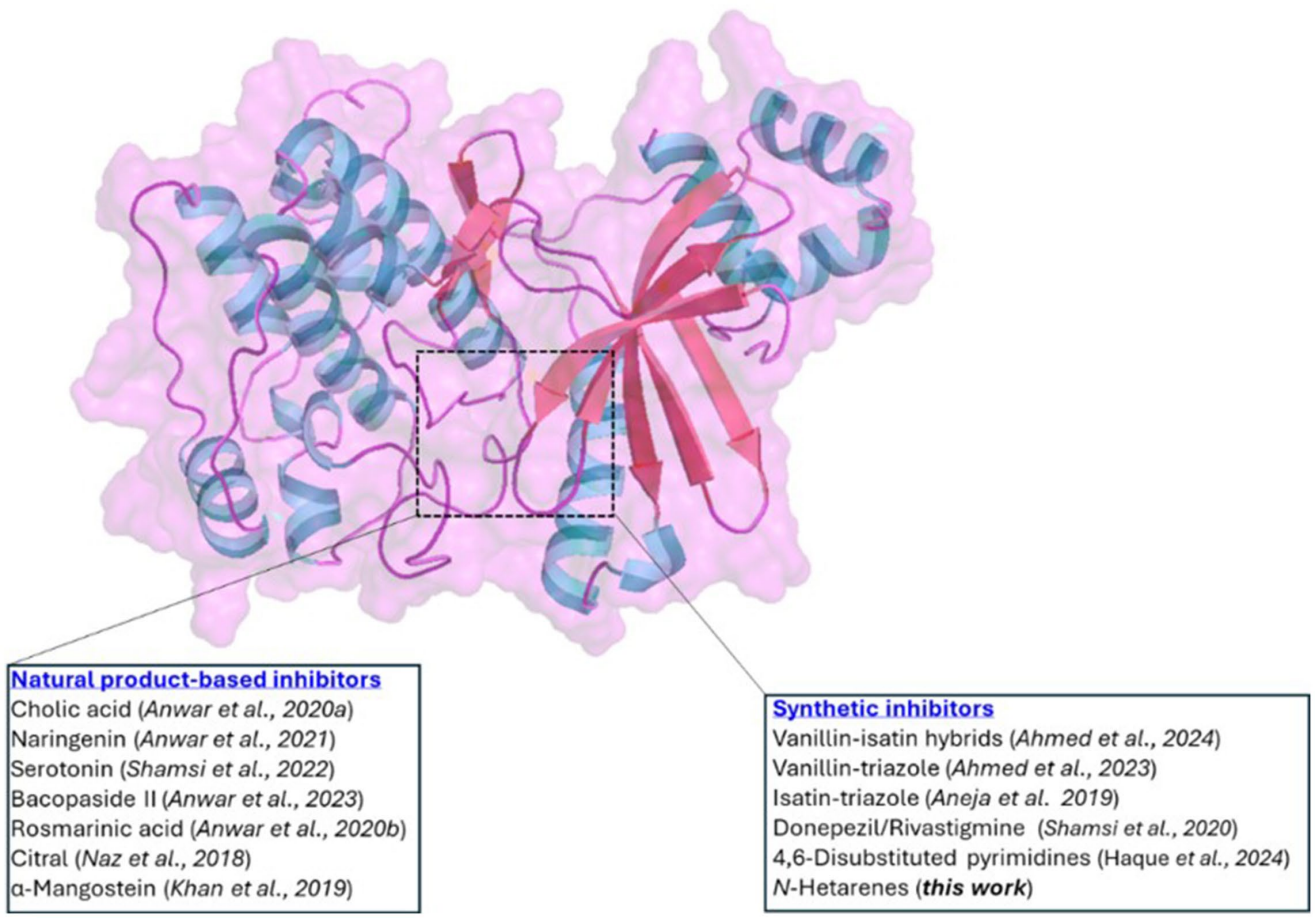
Cartoon diagram illustrating the structure of MARK4. The highlighted area indicates the ATP-binding site, where both natural products and synthetic inhibitors bind.

Protein kinases have emerged as intriguing targets as they are reportedly involved in multiple signaling pathways and play essential roles in diabetes, cancer and neurodegenerative diseases (9, 10). Among kinases, microtubule affinity-regulating kinase 4 (MARK4) has been identified as being overexpressed in various pathological conditions, including AD (11). It is a member of Ser/Thr kinase, which is expressed in brain neurons and phosphorylates toxic tau, whose accumulation causes AD and related disorders (12). It has been recently shown that neuroinflammation and cellular death can be reversed by downregulating the MARK4 signaling system in AD (13). Considering its potential as a drug target, various researchers reported carbocyclic and heterocyclic small molecules as anti-MARK4 compounds (12). Hassan’s research team has conducted extensive studies in MARK4-related diseases and reported a plethora of synthetic and naturally occurring MARK4 inhibitors (Figure 1). Recently, they reported hybrids composed of vanillin-isatin cores with inhibitory concentrations in the micromolar range (IC_50_ = 7.16–8.31 μM) (14). Before this, the same group reported vanillin-triazole conjugates (15) and isatin-triazole hydrazones (16). This group also showed that anti-AD drugs donepezil and rivastigmine may function as multi-target ligands. They found these compounds inhibited the MARK4 enzyme *in vitro* (IC_50_ = 5.3 μM and 6.74 μM for donepezil and rivastigmine, respectively). This suggests that, in addition to their primary use, these drugs may have broader therapeutic implications by modulating multiple targets (17). Other groups also studied different classes of small molecules (18–20) and peptides (12) as anti-MARK4 compounds. Among natural products, the binding affinity and inhibition by cholic acid (21), naringenin (22), serotonin (23), bacopaside II (24), rosmarinic acid (25), citral (26), *α*-mangostein (27) and others (28) have been studied. Recently, we reported a series of 4-(4-(arylsulfonyl)piperazin-1-yl)-6-(thiophen-3-yl)pyrimidine in which we varied arylsulfonyl rings attached to piperazine core and assessed their MARK4 binding using experimental and theoretical tools (29). We found that compounds inhibited MARK4 with an IC_50_ = 7.52 ± 0.33 to 37.99 ± 0.62 μM, and the activity was the function of the electronic nature of the aryl substituents. Motivated by the high druggability of MARK4 and its inhibition by *N*-hetarenes, we report herein the synthesis, characterization, *in-vitro*, and *in-silico* studies of MARK4 inhibitors based on pyrimidine-based small molecules 4-(6-(arylpyrimidin-4-yl)piperazine-1-carboximidamide derivatives **5−10**.

## 2 Materials and methods

### 2.1 General

All chemicals were obtained from Sigma Aldrich and used as received. All reactions/manipulations were carried out under an inert (N_2_) atmosphere. ^1^H- and ^13^C-NMR were collected on Bruker Spectrospin DPX 500 MHz spectrometer (Bruker Analytic GmbH, Berlin, Germany). Splitting patterns are designated as follows: s, singlet; d, doublet; m, and multiplet. Chemical shift values (*δ*) are given in ppm. High-resolution mass spectrometry (HRMS) was performed using an Agilent 1,290 Infinity II UPLC/HRMS instrument equipped with a Q-TOF (UHD Accurate-Mass) and a photodiode array detector. The progress of the reaction was monitored using thin-layer chromatography (TLC) and purification was performed using gravity column chromatography using silica gel (200–400 mesh) and ethyl acetate/hexane as eluent.

### 2.2 Synthesis and characterization

#### 2.2.1 Synthesis of intermediates (3 and 4)

*^t^*Butyl 4-(6-chloropyrimidin-4-yl)piperazine-1-carboxylate **1** was prepared *via* the reaction of 4,6-dichloro pyrimidine and Boc-protected piperazine following the reported method (29). In a 50 mL sealed tube containing mixture of dioxane/H_2_O (8/2), a stoichiometric amount of **1** (1.0 mmol) and different arylboronic acid (**2**, 1.5 equiv.) was added followed by the addition of base (K_2_CO_3_, 6 equiv.). The reaction mixture was purged with nitrogen gas for 2 min, prior adding the Pd(0) catalyst (5 mol%) under a nitrogen atmosphere. The reaction was allowed to be heated at 100°C for 3 h. Upon the completion of the reaction, the solvent was evaporated on a rotary evaporator and the crude product was purified using gravity column chromatography (eluent: EtOAc/Hex = 8/2) yielding *^t^*butyl 4-(6-arylpyrimidin-4-yl)piperazine-1-carboxylate **3**. TFA-induced deprotection in dichloromethane (DCM) yielded compound 4-aryl-6-(piperazin-1-yl)pyrimidine **4**.

#### 2.2.2 Synthesis of 4-(6-(arylpyrimidin-4-yl)piperazine-1-carboximidamides (**5**−**10**)

To 50 mL of the round bottom flask containing 10 mL of DCM, compound **4** (1.0 mmol) was added while stirring. This is followed by portion-wise addition of 1*H*-pyrazole-1-carboximidamide (1.5 equiv.) and Et_3_N (1.2 equiv.) at 0°C. The reaction was stirred at room temperature for 3−12 h. Following the completion of the reaction, the reaction mixture was diluted with diethyl ether. The resulting precipitate was filtered and washed with several portions of diethyl ether and dried to obtain 4-(6-(arylpyrimidin-4-yl)piperazine-1-carboximidamides **5−10** in good to excellent yield.

##### 2.2.2.1 4-(6-(p-Tolyl)pyrimidin-4-yl)piperazine-1-carboximidamide **(5)**

Off-white solid; yield = 73%, m.p. = 170°C; ^1^H-NMR (500 MHz, DMSO-d_6_) *δ* 8.56 (s, 1H), 8.02 (d, *J* = 8.1 Hz, 2H), 7.52 (s, 3H), 7.30 (d, *J* = 8.0 Hz, 2H), 7.25 (s, 1H), 3.81 (s, 4H), 3.57 (s, 4H), 2.31 (s, 3H); ^13^C- NMR (126 MHz, DMSO-*d*_6_) *δ* 162.39, 158.30, 156.73, 140.73, 134.71, 129.79, 127.23, 98.53, 44.68, 42.72, 21.34.

##### 2.2.2.2 4-(6-(3,4-Dichlorophenyl)pyrimidin-4-yl)piperazine-1-carboximidamide (**6**)

Light yellow solid; yield = 68%, m.p. = 152°C; ^1^H-NMR (500 MHz, DMSO-d_6_) *δ* 8.61 (s, 1H), 8.44 (d, *J* = 2.1 Hz, 1H), 8.20 (dd, *J* = 8.4, 2.1 Hz, 1H), 7.84–7.71 (m, 4H), 7.48 (s, 1H), 3.95–3.77 (m, 4H), 3.60 (t, *J* = 5.3 Hz, 4H). ^13^C-NMR (126 MHz, DMSO-d_6_) δ 162.50, 159.54, 158.49, 156.95, 138.22, 133.34, 132.15, 131.33, 129.01, 127.41, 99.55, 44.75, 42.94. HRMS-CI (m/z): [M + H]^+^ calcd for C_15_H_17_Cl_2_N_6_, 351.0892 amu; found, 351.1643 amu.

##### 2.2.2.3 4-(6-(3-Chloro-4-fluorophenyl)pyrimidin-4-yl)piperazine-1-carboximidamide **(7)**

Yellowish solid; yield = 74%, m.p. = 160°C; ^1^H-NMR (500 MHz, DMSO-d_6_) *δ* 8.60 (s, 1H), 8.42 (dd, *J* = 7.3, 2.2 Hz, 1H), 8.29–8.16 (m, 2H), 7.79 (d, *J* = 13.7 Hz, 1H), 7.54 (t, *J* = 8.9 Hz, 1H), 7.46 (s, 1H), 7.03 (s, 1H), 3.91–3.79 (m, 4H), 3.60 (t, *J* = 5.0 Hz, 4H). ^13^C-NMR (126 MHz, DMSO-d_6_) δ 162.48, 159.71, 159.59, 158.44, 158.37, 157.87, 156.96, 135.42, 135.39, 129.42, 128.22, 128.16, 120.58, 120.44, 117.69, 117.53, 111.13, 102.51, 99.29, 53.41, 44.76, 44.53, 41.81. HRMS-CI (m/z): [M + H]^+^ calcd for C_15_H_17_ClFN_6_, 335.1187 amu; found, 335.1261 amu.

##### 2.2.2.4 4-(6-(4-(Trifluoromethyl)phenyl)pyrimidin-4-yl)piperazine-1-carboximidamide **(8)**

Yellowish solid; yield = 65%, m.p. = 178–180°C; ^1^H-NMR (500 MHz, DMSO-d_6_) *δ* 8.66 (d, *J* = 1.0 Hz, 1H), 8.41 (d, *J* = 8.1 Hz, 2H), 7.87 (d, *J* = 8.2 Hz, 2H), 7.75 (s, 2H), 7.51 (s, 2H), 3.94–3.77 (m, 4H), 3.60 (dd, *J* = 6.7, 3.9 Hz, 4H). ^13^C-NMR (126 MHz, DMSO-d_6_) *δ* 162.51, 160.63, 158.59, 156.91, 141.63, 130.53, 128.15, 126.01, 100.10, 44.73, 42.89. HRMS-CI (m/z): [M + H]^+^ calcd for C_16_H_18_F_3_N_6_, 351.1545 amu; found, 351.1005 amu.

##### 2.2.2.5 4-(6-(benzo[*b*]thiophen-2-yl)pyrimidin-4-yl)piperazine-1-carboximidamide (**9**)

Yellowish solid; yield = 78%, m.p. = 168°C; ^1^H-NMR (500 MHz, DMSO-d_6_) *δ* 8.37 (s, 1H), 7.79 (d, *J* = 15.7 Hz, 8H), 7.03 (s, 1H), 3.76 (s, 4H), 3.57 (s, 4H).^13^C-NMR (126 MHz, DMSO-d_6_) *δ* 162.48, 159.59, 158.38, 156.94, 102.51, 44.53, 44.32, 42.98. HRMS-CI (m/z): [M + H]^+^ calcd for C_17_H_19_N_6_S, 339.1392 amu; found, 339.1495 amu.

##### 2.2.2.6 4-(6-(4-(Trifluoromethoxy)phenyl)pyrimidin-4-yl)piperazine-1-carboximidamide **(10)**

Off-white solid; yield = 70%, m.p. = 172–175°C; ^1^H-NMR (500 MHz, DMSO-d_6_) *δ* 8.63 (s, 1H), 8.31 (d, *J* = 8.9 Hz, 2H), 7.61–7.47 (m, 5H), 7.43 (s, 1H), 3.86 (t, *J* = 5.2 Hz, 4H), 3.58 (s, 4H). ^13^C-NMR (126 MHz, DMSO-*d*_6_) δ 162.39, 160.93, 158.39, 156.78, 148.46, 136.72, 129.43, 120.73, 102.36, 99.80, 53.94, 45.05. HRMS-CI (m/z): [M + H]^+^ calcd for C_16_H_18_F_3_N_6_O, 367.1494 amu; found, 367.1577 amu.

### 2.3 Biological studies

#### 2.3.1 Enzyme inhibition assay

Purified MARK4 was used to conduct ATPase inhibition experiment following the reported method (18, 30). The concentration of the purified protein used was 5 μM, the ligand concentration was 0–10 μM and the substrate (ATP) was 200 μM. The ligand concentration was increased gradually against a given concentration of MARK4. The experiment involved combining MARK4 with freshly prepared ATP, resulting in a final reaction mixture volume of 100 μL. This mixture was incubated for 1 h at 25°C. This is followed by the addition of Malachite green solution (200 μL) to quench the reaction. The samples were allowed to incubate at room temperature for color development. Finally, the reaction mixture (100 μL) was filled into 96-well plates and analyzed using a multiscan ELISA reader (*λ* = 620 nm). All experimental reactions were conducted in triplicate to ensure statistical reliability.

#### 2.3.2 Fluorescence quenching assay

A fluorescence-based binding assay was conducted to evaluate the binding efficiency of ligands. During the titration, the concentration of the pure MARK4 protein was kept constant while increasing concentrations of the ligand (31). The emission data were collected using a Jasco FP-6200 spectrofluorimeter (Japan), with a slit width of 10 nm and medium sensitivity. The samples were excited at 280 nm, and the emission spectra were recorded. Blank subtracted spectra are reported, and each measurement was performed in triplicate. For fluorescence values, we took the inner filter effect into account (32). The binding constant (*K*) was calculated using the Stern-Volmer (SV) (Equations 1, 2).


(1) $$\frac{F_{0}}{F}=1+K_{sv}\left[C\right]$$

(2) $$\log\frac{F_{0}-F}{F}=\mathrm{logK}+n\log\left[C\right]$$

Where *F*_0_ denotes the fluorescence intensity of the protein in the absence of ligands, whereas *F* indicates the fluorescence intensity when ligands are present. *K* represents the binding constant, n indicates the number of binding sites, and C refers to the concentration of the ligand.

### 2.4 Computational details

The computational work was performed on a Dell Precision 7,920 workstation with 20 Intel Xeon Silver cores, 128 GB RAM, and an NVIDIA Quadro RTX 8000 GPU with 32 GB of memory. Density functional theory (DFT) calculations were performed on a Tyrone workstation equipped with an Intel® Xeon® Gold 5218R CPU @ 2.10GHz (40 cores) and an NVIDIA Quadro P2200 GPU (GP106GL).

#### 2.4.1 Selection of target and reference molecules

The MARK4 protein (PDB ID: 5ES1) was chosen as the target in this study (33). The 3D structure of MARK4 in complex with the ligand *N*-(6-amino-2,2-difluorocyclohexyl)-5-ethyl-4-(6-(trifluoromethyl)pyrazolo[1,5-a]pyrimidin-3-yl)thiophene-2-carboxamide (5RC) was retrieved from the Protein Data Bank (PDB) and saved in .pdb format.

#### 2.4.2 Molecular docking

##### 2.4.2.1 Active site prediction and ligand preparation

Missing residues were modelled as reported previously (29). The active site of the MARK4 protein (PDB ID: 5ES1) was identified and analyzed using PDBsum (34) and CASTp (35) tools. The structure of the ligands (**5**–**10** and 5RC) were drawn, optimized and converted to .pdbqt format for molecular docking using AutoDock 4.2.6 (36) and MGLTools 1.5.7 (37).

##### 2.4.2.2 Protein preparation and grid generation

The 3D structure of the receptor was prepared by adding missing hydrogen atoms, including polar hydrogens, removing water molecules and heteroatoms. Energy minimization was carried out, and atomic charges were assigned (38). The protein structure was saved in the .pdbqt format. A docking grid around the MARK4 binding site was generated, centered on the co-crystallized inhibitor, covering the binding and active site residues. A grid box of dimensions X = 70 Å, Y = 88 Å, and Z = 84 Å axes was generated with a default grid spacing of 0.37 Å to ensure precise ligand docking within the active site.

##### 2.4.2.3 Docking studies

The ligands (**5**–**10** and 5RC) were docked into the MARK4 active site to assess binding affinity. Each ligand received a score based on predicted binding free energy, where lower scores indicated stronger interactions (39). The resulting output files were visualized using PyMOL to compare binding orientations (40).

#### 2.4.3 Molecular dynamics (MD) simulation

MD simulation is a powerful technique used to study protein-ligand complexes, providing detailed insights into their dynamic behavior, structural stability, and interactions under physiological conditions. We utilized the Desmond module of Schrödinger for the simulations (41, 42). Using the system builder module, the ligand-protein complex was first selected and prepared for the MD simulation. An orthorhombic simulation box of dimensions 10 × 10 × 10 Å was created, and the SPC (Simple Point Charge) model was chosen as the solvent model (43). The OPLS3 force field was employed to represent the interactions within the system (44, 45). To mimic physiological conditions, sodium (Na^+^) and chloride (Cl^−^) ions were added to the system (0.15 M). Following this, energy minimization was performed for 100 picoseconds (ps) to relax the model into a local energy minimum. The minimized structure was then subjected to MD simulation for 500 nanoseconds (ns) under an NPT ensemble, which maintains constant temperature (300 K) and pressure (1.01325 bar) maintained using the Nosé-Hoover thermostat and Martyna-Tobias-Klein barostat, as implemented in the Desmond module (46–48). The results of the simulation were analyzed by comparing root mean square deviation (RMSD), root mean square fluctuations (RMSF) and secondary structure elements (SSEs).

#### 2.4.4 Drug-likeness, bioavailability, and toxicity prediction

The drug-likeness, absorption and distribution and toxicity of the compounds under investigation were predicted by SwissADME (49), pKCSM (50) and Qikprop (51, 52) tools.

#### 2.4.5 Density functional theory (DFT) studies

Geometry optimization, chemical reactivity descriptors, frontiers molecular orbitals, energy and other properties of the compounds were computed by DFT (B3LYP/6-31G*) using Spartan 24 software (53).

## 3 Results and discussion

### 3.1 Synthesis and characterization of 4-(6-(arylpyrimidin-4-yl)piperazine-1-carboximidamides (**5**–**10**)

**Figure 2:**
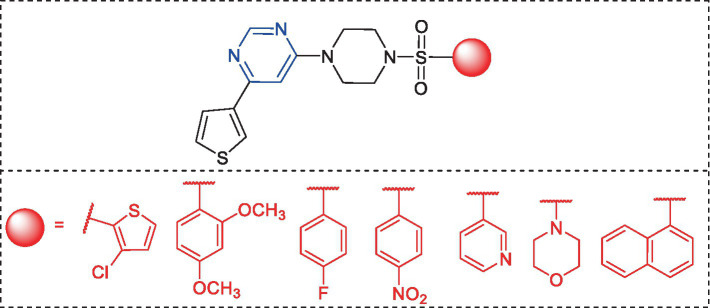
Chemical structure of the 4,6-disubstituted pyrimidines with MARK4 inhibitory activity (29).

**SCHEME 1:**
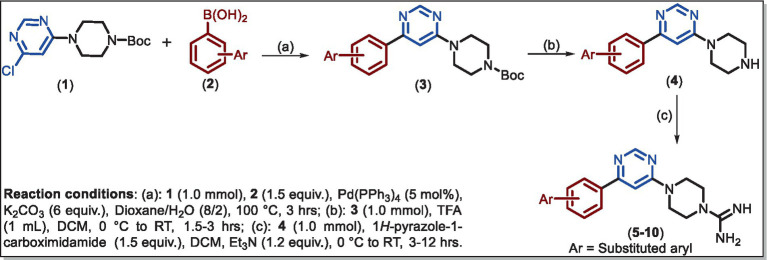
Synthetic scheme for the intermediate (**3–4**) and final (**5**–**10**) compounds.

Pyrimidine and piperazine are privileged scaffolds in drug discovery and are part of various drugs, including small-molecule kinase inhibitors (54). We reported on some 4,6-disubstituted pyrimidines in which different arylsulfonyl groups were attached through piperazine fragments (Figure 2) (29). *In-vitro* results suggested enzyme inhibition in the micromolar range, indicating a fundamental affinity of these compounds to MARK4. Following the same line of reasoning, a new series of derivatives is presented in this paper. We speculate that the replacement of 4-(4-(arylsulfonyl)piperazin-1-yl) flank by a more basic moiety and thienyl by different aromatic cores with varying electronic nature should lead to molecules with improved solubility and biological properties. Motivated by this, we report the synthesis, chemical characterization, and biological characterization of 4-(6-(arylpyrimidin-4-yl) piperazine-1-carboximidamides **5–10** (Scheme 1).

Compound **1** was obtained by a selective S_N_Ar reaction following the reported protocol (29) and the data are given in Supplementary Figure S1. Suzuki coupling of **1** with commercially available different arylboronic acid **2** yielded *^t^*butyl 4-(6-arylpyrimidin-4-yl)piperazine-1-carboxylate **3** in good yields. Then, the Boc group is removed by TFA in DCM and the resulting product **4** reacts with 1*H*-pyrazole-1-carboximidamide to provide **5–10** in 65–78% yields.

**Figure 3:**
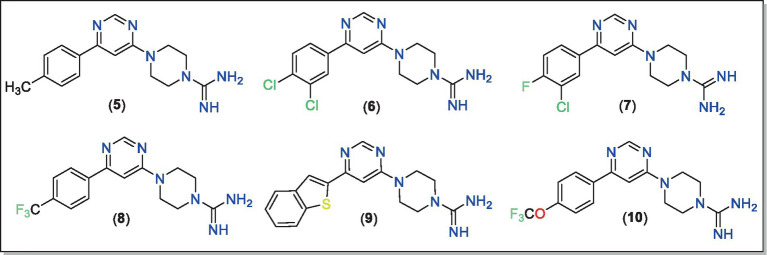
Chemical structures of the final compounds (**5–10**) studied in this work.

In the ^1^H-NMR spectra of **5–10**, expected signals due to aryl, pyrimidine, piperazine and other motifs confirmed the structure of the compounds (Supplementary Figures S2–S7). For example, two singlets integrating for two hydrogens of the pyrimidine ring appeared at *δ* 8.37–8.66 ppm and δ 7.03–7.52 ppm (29, 55). The signals resonating at δ 3.57–3.60 ppm and δ 3.76–3.95 ppm were attributed to the piperazine fragment. In compound **5,** the signals due to three protons of methyl were observed at δ 2.31 ppm. In the ^13^C-NMR spectra, signals of methylene carbon of piperazine were observed in the regions δ 41.81–42.98 ppm and δ 44.53–45.05 ppm. In addition, signals at δ 162.39–162.51 for *C**_(Ar)_*–N, δ 158.30–159.71 ppm for *C* =N and δ 21.34 ppm for *C*H_3_ further supported the chemical structure. High-resolution mass spectra (Supplementary Figure S8) showed molecular ion peaks for all compounds, confirming the formation of final products (Figure 3).

### 3.2 Biological assay

#### 3.2.1 Enzyme inhibition assay

**Figure 4:**
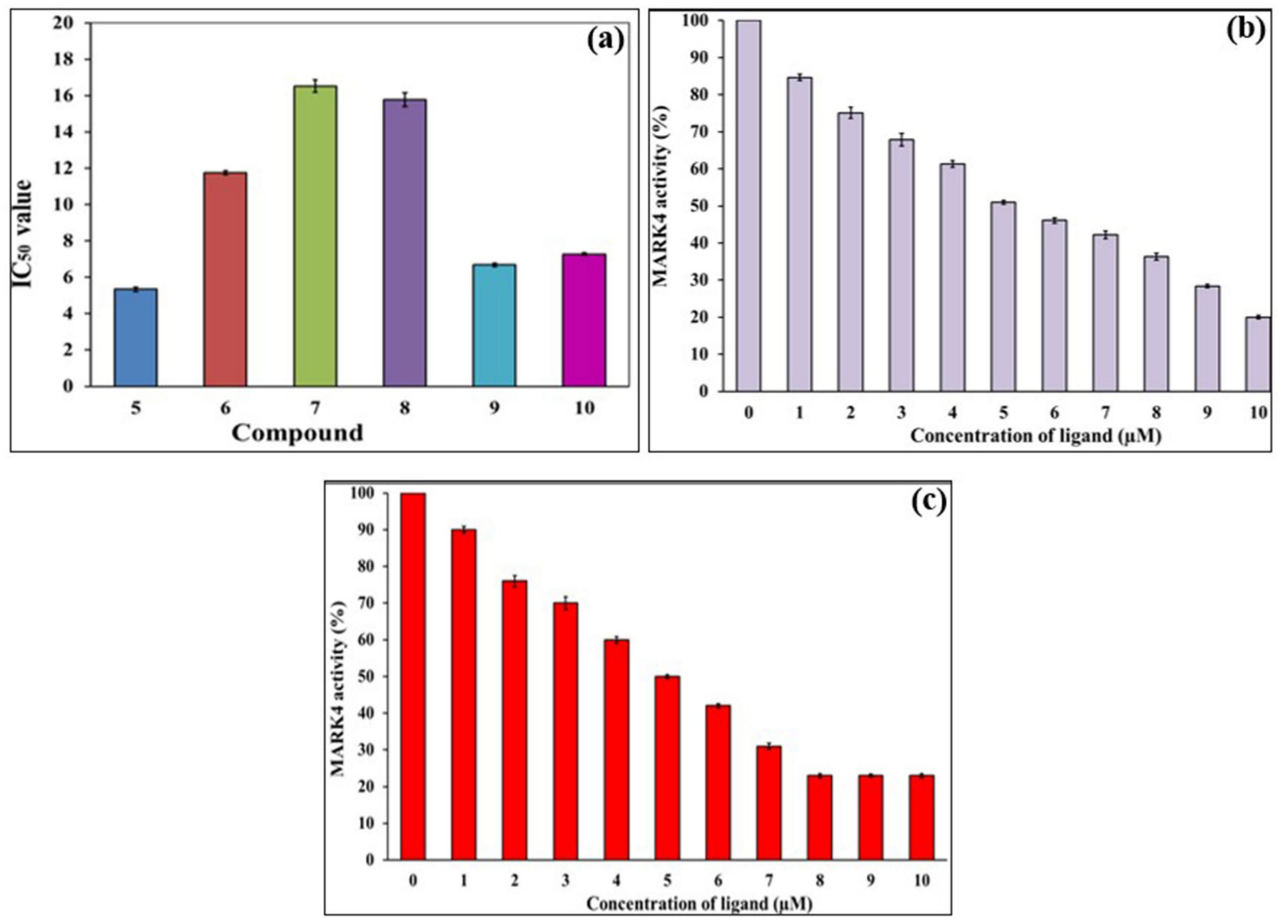
ATPase inhibition assay of compounds **5**–**10** against MARK4 **(a)**, Changes in the % activity of MARK with increasing concentration of **5 (b)** and **9 (c)**.

Six-membered pyrimidines are a privileged scaffold in drug discovery, especially in neurodegenerative diseases (56). While 4,6-disubstituted pyrimidines have been extensively studied in the past against AD-related targets such as AchE (57), tau (58), *β*-amyloid aggregation (59) and monoamine oxidase (60), limited studies have been carried out against MARK enzymes. We recently demonstrated that the MARK4 inhibitory activity depended on the substitution pattern of 4-(4-(arylsulfonyl)piperazin-1-yl) flank in 4,6-disubstituted pyrimidine derivatives (29). To assess the inhibitory potentials of compounds **5**–**10**, an ATPase inhibition assay against MARK4 was carried out following the reported method (18, 30). MARK4, currently does not have an FDA-approved inhibitor. However, numerous compounds are progressing through the pre-clinical stages of development. For this study, we chose Rosmarinic acid (IC_50_ ~ 6.20 μM) as a reference inhibitor (25). Its potent bioactivity makes it a valuable reference point in exploration of potential MARK4 inhibitors. The results of the kinase activity assay are compiled in Table 1 and shown in Figures 4a–c. Regarding the activity, compound **5** (IC_50_ = 5.35 ± 0.22 μM) showed the best activity, followed by **9** (IC_50_ = 6.68 ± 0.80 μM). Notably, neither of these top-performing compounds contains any halogen substituents. In contrast, compounds **6**, **7**, and **8**, which feature two or more halogen atoms attached to their aromatic rings, displayed lower activity, with IC_50_ values of 11.76 ± 1.19 μM, 16.53 ± 1.71 μM, and 15.78 ± 0.92 μM, respectively. Furthermore, compound **10**, which possesses a trifluoromethoxy group at the 4-position, showed intermediate activity with an IC_50_ = 7.28 ± 0.49 μM, situating it between the two extremes observed. To further elucidate the underlying reasons for these activity trends, we performed extensive *in-silico* studies (*vide-infra*). Following this, we evaluated the effects of compounds **5** and **9** across a range of concentrations (0 to 10 μM). Notably, we observed a dose-dependent activity pattern: as the concentration of these compounds increased, there was a corresponding decrease in their activity. This reduction in MARK4 activity is noteworthy and suggests that the behavior of compounds **5** and **9** is comparable to, or even superior to, that of several small molecules derived from both natural sources (25, 26) or synthetic origin (14). On the contrary, when we conducted assays targeting pyruvate dehydrogenase kinase 3 (PDK3), these compounds displayed a weaker affinity (*data not included*). Such selectivity towards MARK4 of the azole-based compounds is already known (16). Therefore, compounds **5** and **9** stand out as representatives of a novel class of MARK4 inhibitors.

**Table 1:** The 50% inhibitory concentration (IC_50_) of compounds **5**–**10** against MARK4.

**Code**	**5**	**6**	**7**	**8**	**9**	**10**
IC_50_ (μM)	5.35 ± 0.22	11.76 ± 1.19	16.53 ± 1.71	15.78 ± 0.92	6.68 ± 0.80	7.28 ± 0.49

#### 3.2.2 Fluorescence quenching assay

**Figure 5:**
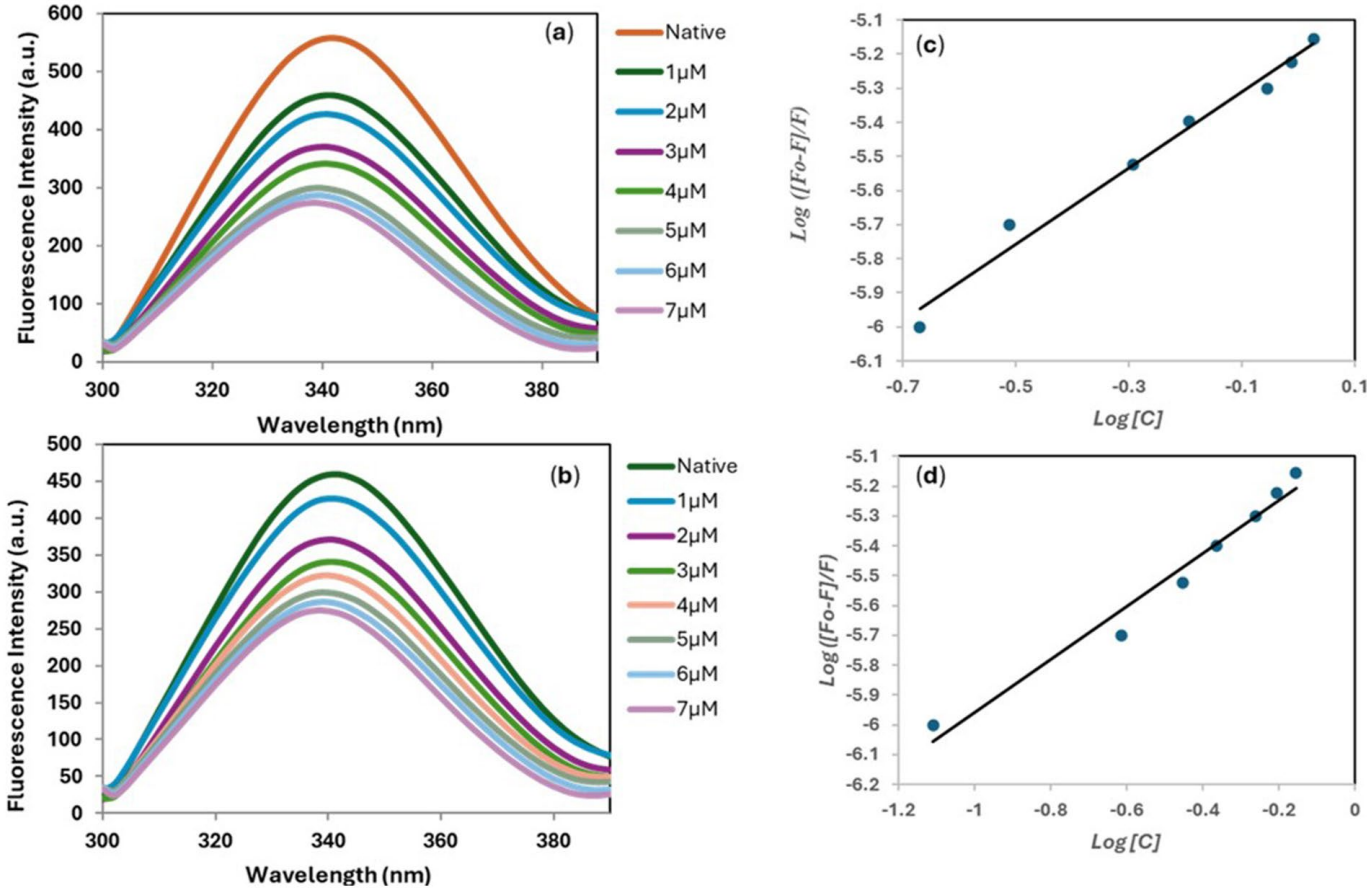
Fluorescence emission spectra of MARK4 in the absence and presence of **5 (a)** and **9 (b)**. Modified Stern-Volmer plot of **5-**MARK4 **(c)** and **9-**MARK4 **(d)** complexes.

The presence of aromatic amino acid (tryptophan) in the MARK4 protein results in a characteristic fluorescence emission near 344 nm when excited at 280 nm (25). To get deeper insight into the interactions between the **5**-MARK4 and **9**-MARK4 complexes, we conducted fluorescence binding studies and determined the binding constant (*K*). As we gradually introduced increasing concentrations of compound **5** (0–7 μM) to the native MARK4, we observed a notable decrease in the fluorescence intensity (Figures 5a,b). This shift was accompanied by a slight blue shift in the emission maximum, approximately 5–7 nm. A similar pattern emerged with the **9**-MARK4 complex, further supporting the notion that the ligand is effectively binding to the receptor. A decrease in intensity is also indicative of a stable complex formed between the ligand and protein (61). By employing a modified Stern-Volmer equation, we calculated the binding constants (Figures 5c,d), which were found to be 1.5 ± 0.51 × 10^5^ M^−1^ for **5** and 1.14 ± 0.26 × 10^5^ M^−1^. These binding constants highlight a strong affinity between the compounds and the MARK4 protein.

### 3.3 Computational studies

#### 3.3.1 Molecular docking

**Figure 6:**
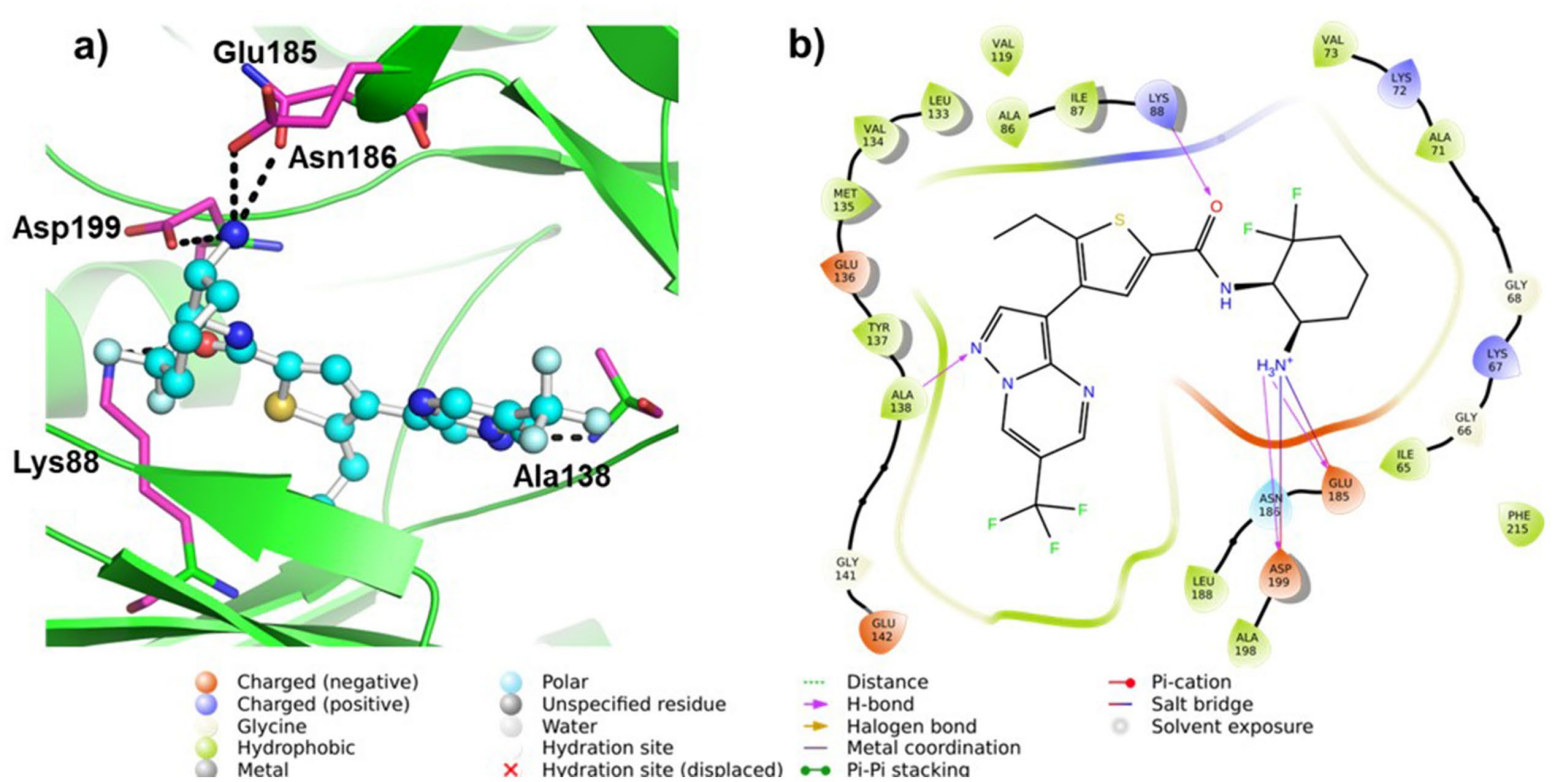
**(a)** 3D and **(b)** 2D plots of the interaction of MARK4 (PDB: 5ES1) complex containing ligand 5RC.

**Figure 7:**
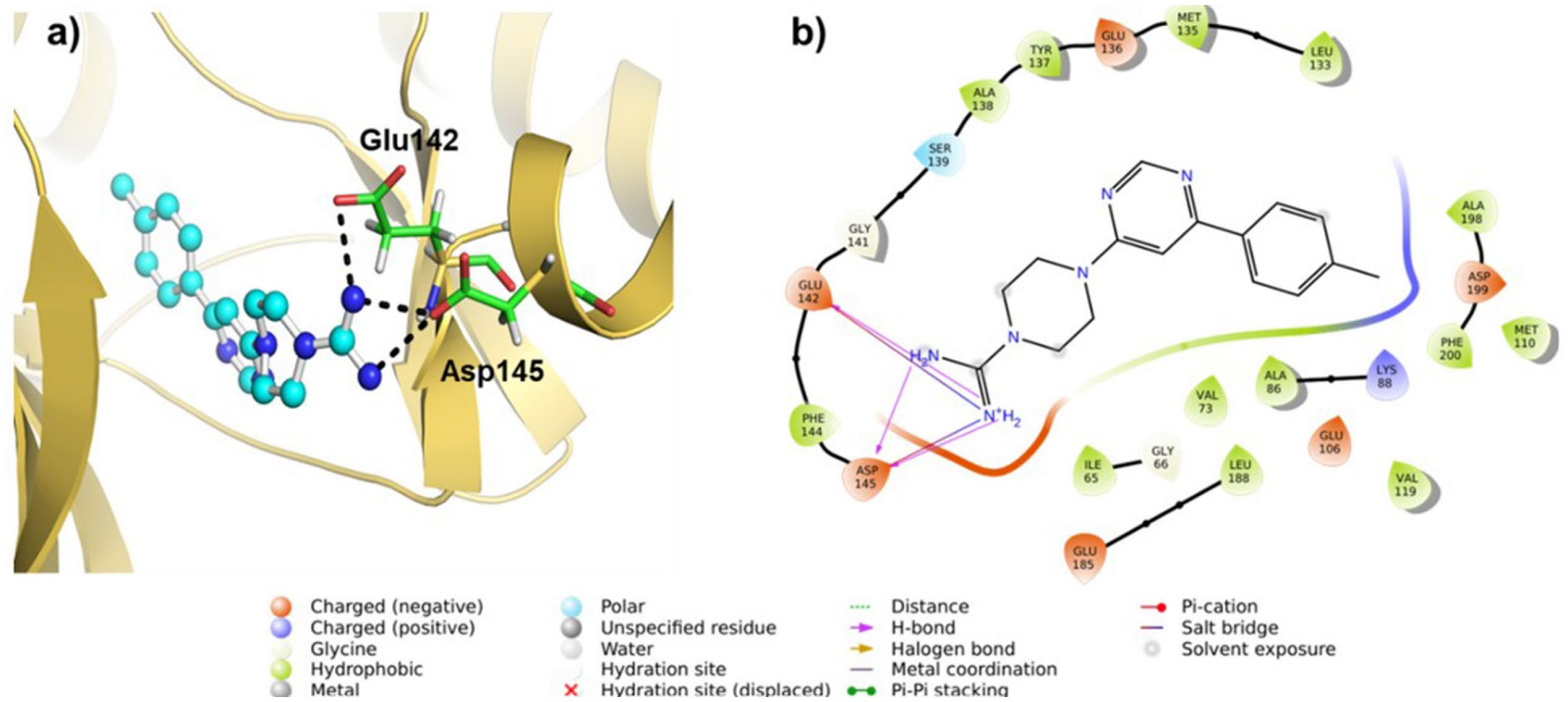
**(a)** 3D and **(b)** 2D plots of the interaction of MARK4 (PDB: 5ES1) complex containing ligand **5**.

We conducted molecular docking studies to underpin the role of different functionalities on their biological activity and elucidate the binding mode and strength between MARK4 and ligands. The ligand (5RC) was used as the reference compound. Table 2 collects the docking score and binding residues of MARK4 complexed with 5RC and compounds **5**–**10**. All compounds, including the inhibitor 5RC, are targeted in the ATP binding pocket and bind to the deep binding cavity of MARK4 (62). The docking score of the compounds was higher than the ligands like huperzine (19). The residues responsible for binding the ligands to the active site of MARK have been reported to be involved in the binding of ATP (a pyrimidine-based molecule) and other inhibitors (62). Figures 6, 7 displays the 2D/3D docking poses and interacting residues between 5RC or **5** and the MARK 4 receptor, while the other compounds are given in Supplementary Figures S9, S10. 5RC formed H-bond with Lys88, Ala138, Glu185, Asn186, and Asp199 of the active site of MARK4 (Figure 6). Binding free energy (ΔG) for MARK4 complexed with 5RC was −8.0 kcal/mol, indicating reasonable binding affinity. However, an increase in affinity (ΔG = −8.4 kcal/mol) can be noted for the complex containing **5**. Indeed, the NH_2_ group of guanidine facilitated the formation of H-bonds with the backbone amino acids Glu142 and Asp145 (Figure 7). Such residues have also been reported to be involved in other heterocyclic compounds (62). In addition, salt bridge interaction was also observed between the ligand and the side chains (Glu142 and Asp145), enhancing the ligand’s affinity for the kinase domain. A decrease in affinity (ΔG = −7.7 kcal/mol) can be seen when the tolyl ring attached to the pyrimidine was replaced by 3,4-dichlorophenyl in **6**. It formed one only H-bond with Ser139 of MARK4. However, when the chloro was replaced with F (compound **7**), an increase in the docking score (ΔG = −8.2 kcal/mol) was noted despite having one H-bond with Asp142 residue. However, the omission of a second chlorine and the introduction of a 4-trifluoromethyl (compound **8**) or 4-trifluoromethoxy (compound **10**) were found to be detrimental, as indicated by reduced docking score (ΔG = −7.8 kcal/mol for both), despite having one H-bond with Tyr137 and Lys67, respectively. Compound **9**, containing an electron-rich benzothienyl core, demonstrated affinity like compound **8** (ΔG = −7.9 kcal/mol) and formed one H-bond with residue Tyr137. Summarily, this study found that among the screened compounds, **5** is the most promising candidate. It is also notable that the guanidine moiety attached to piperazine participated in H-bonding. A change in the aryl ring only affected the orientation of ligands of the molecules.

**Table 2:** Docking scores and binding residues of compounds **5**–**10** and 5RC.

Parameters	Ligands						
	5RC	5	6	7	8	9	10
Binding energy (ΔG, kcal/mol)	–8.0	–8.4	–7.7	–8.2	–7.8	–7.9	–7.8
Binding residues (H-Bond only)	Lys88, Ala138, Glu185, Asn186, and Asp199	Glu142, and Asp145	Ser139	Asp142	Tyr137	Tyr137	Lys67

#### 3.3.2 MD simulation

**Figure 8:**
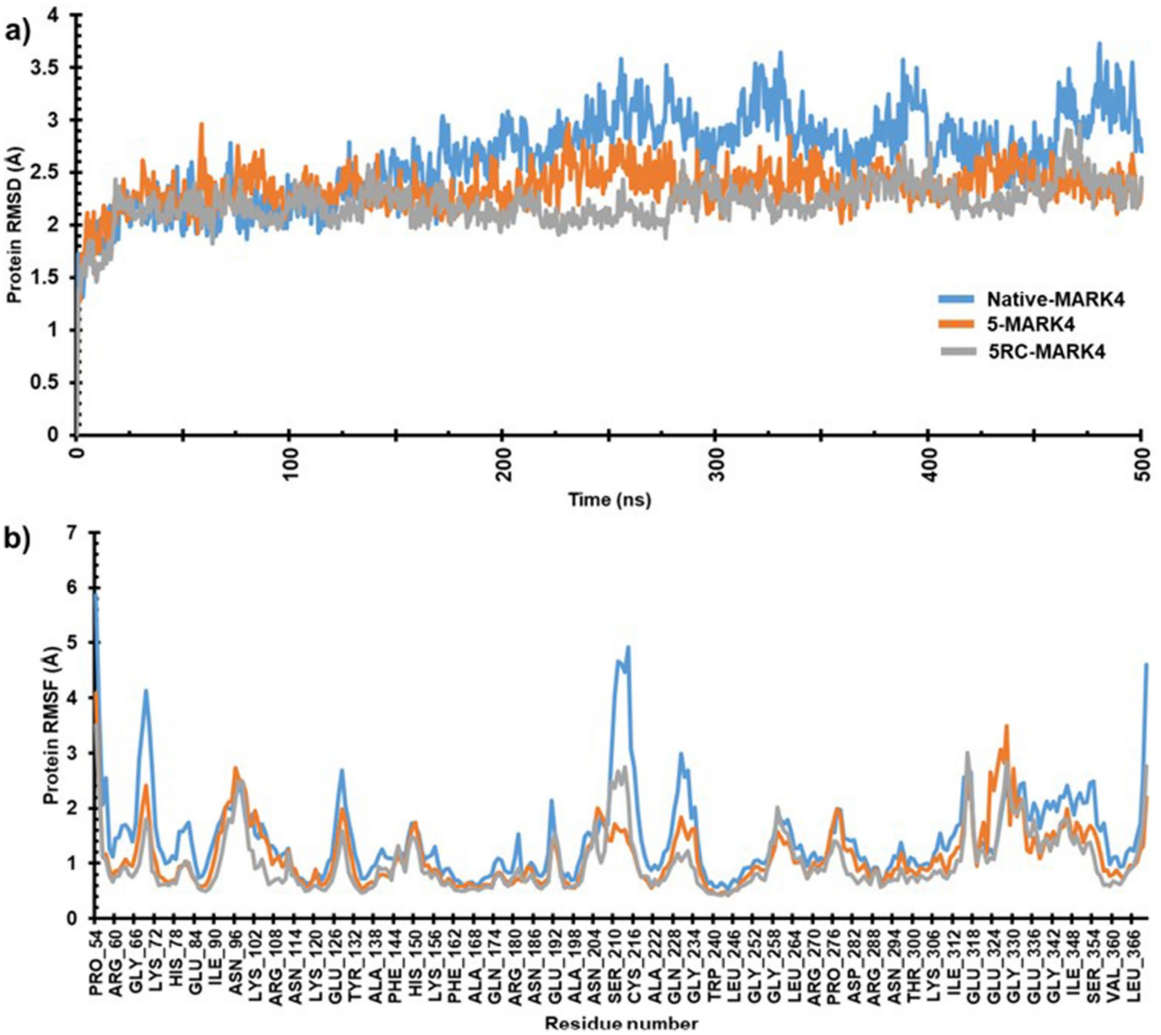
**(a)** RMSD and **(b)** RMSF plots of native MARK4 and its complexes with **5** and 5RC.

The MD simulations of native and MARK4 complex with compound **5** were conducted for 500 ns each. Both systems achieved equilibrium within the first 50 ns. The initial trajectories up to 150 ns displayed only minor fluctuations. However, after 150 ns, the native MARK4 exhibited a progressively increasing RMSD, indicating a deviated and less stable trajectory. In contrast, the **5**-MARK4 complex demonstrated stable behavior beyond 150 ns, with the trajectory showing minimal fluctuations, suggesting that compound **5** stabilizes the MARK4 structure during the simulation. The average RMSD values for the entire simulation were 2.64 Å for the native, 2.35 Å for the **5**-MARK4 complex, and 2.30 Å for the cocrystal ligand 5RC (Figure 8a). This further supports the conclusion that compound **5** enhances the structural stability of MARK4. To assess the flexibility of individual amino acid residues during the simulation, RMSF plots of the native, 5-MARK4 complex and cocrystal ligand 5RC were analyzed (Figure 8b). A significant fluctuation can be seen in the residues Gly66-Lys72, Ser210-Cys216, Gln228-Gly234 of the native MARK4, leading to RMSF values reaching ~6 Å with an average RMSF of 1.46 Å. The residue between Lys67 to Ala71 which forms the roof of the catalytic cleft, is already known for its flexibility (62). When complexed with compound **5**, the fluctuation across these residues reduced, indicating the stability of MARK4 in the presence of the ligand. A low average RMSF (1.14 Å) is an indication that the protein was stable throughout the MD simulation. Similarly, the average RMSF of (1.13 Å) for the cocrystal ligand 5RC is almost like the compound **5**.

**Figure 9:**
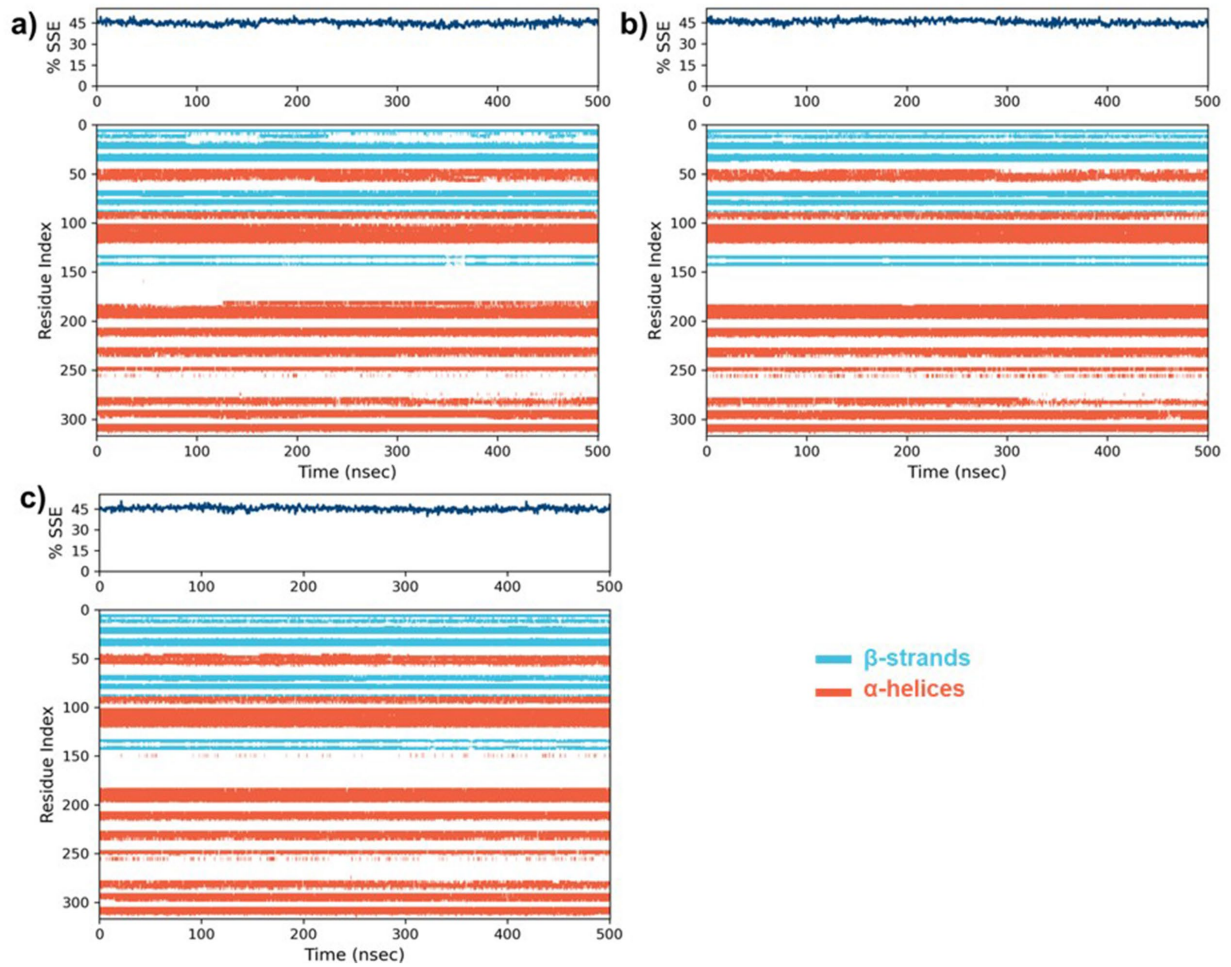
The percentages of secondary structure elements (SSE) along with the residue index for **(a)** the native MARK4 protein, and its complexes with **(b)** compound **5** and **(c)** the co-crystal ligand 5RC.

Following the completion of the RMSD and RMSF analyses, we assessed the secondary structure elements (SSE), with particular emphasis on *α*-helices and *β*-strands (Figure 9). In the case of the native protein, 31.97% of the residues formed α-helix while 13.11% formed β-strands with a total of SSE content 45.08%. A slight drop in α-helix (31.53%) with an increase in β-strand (14.09%) and total SSE (45.61%) was noted for the **5**-MARK4 complex. Similarly, the 5RC complex (31.70%) of the residues formed α-helix while (13.64%) formed β-strands with a total SSE content of 45.34%. This indicates that while the secondary structure of MARK4 remains largely conserved in the presence of compound **5**, there is a slight increase in β-strand content, indicating a subtle stabilization of the secondary structure during the MD simulation.

#### 3.3.3 Drug-likeness, bioavailability, and toxicity prediction

A widely accepted methodology for predicting the potential success of new drug candidates involves the evaluation of their drug-likeness, bioavailability, and toxicity through the use of *in-silico* tools (63). This evaluation also helps determine how promising a drug candidate may be for further development and eventual clinical use. In literature, various tools have been reported that are used to estimate these properties (64, 65). In the present work, we computed these features using SwissADME (49), Qikprop (51, 52), and pKCSM (50) tools (Tables 3–5).

**Table 3:** Calculated drug property data of **5–10** and reference compound 5RC (49).

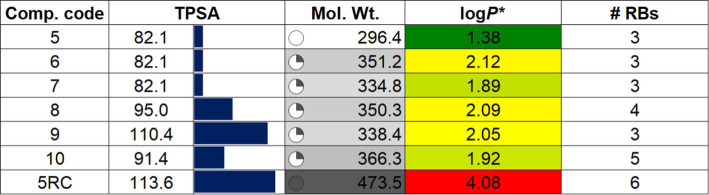

Lighter colors in the property metrics indicate enhanced drug-likeness.

**Table 4:** The drug-likeness properties of **5–10** computed with QikProp.

Code #	#rotor	CNS	Dipole	SASA	FOSA	FISA	PISA	Volume	HBD	HBA	RO5/RO3
**5**	2	–1	4.35	587.96	234.13	140.28	213.55	1007.32	3	4.5	0/0
**6**	2	–1	6.05	601.21	145.86	138.94	186.64	1032.15	3	4.5	0/0
**7**	2	–1	6.16	587.64	146.49	138.94	192.19	1005.02	3	4.5	0/0
**8**	2	–1	6.19	607.94	146.14	138.53	205.64	1044.87	3	4.5	0/0
**9**	1	–1	2.69	613.84	148.02	138.80	290.71	1057.86	3	4.5	0/0
**10**	3	–1	5.71	619.45	146.70	138.84	211.69	1066.59	3	4.5	0/0

**Table 5:** The ADMET properties of **5–10** computed with QikProp.

Code #	QPpolrz	QPlog PC16	QPlog Poct	QPlog Pw	QPlog Po/w	QPlog S	CIQP logS	QPlog HERG	QPP Caco	QPlog BB	QPPM DCK	QPlog Kp	QPlog Khsa	%HOA
**5**	34.91	10.69	18.49	11.93	2.64	−4.79	−4.12	−5.35	463.09	−0.93	215.27	−5.22	0.27	90.13
**6**	35.65	11.64	19.56	11.81	3.26	−5.58	−5.19	−5.31	476.85	−0.61	1141.92	−5.29	0.33	94.00
**7**	34.62	10.72	19.15	11.81	3.03	−5.27	−4.86	−5.26	476.80	−0.65	889.90	−5.27	0.27	92.64
**8**	36.34	10.22	19.781	11.95	3.32	−5.64	−5.17	−5.44	481.04	−0.64	988.96	−5.22	0.36	94.39
**9**	38.31	11.97	19.83	12.74	3.18	−5.55	−5.11	−5.86	478.25	−0.79	352.35	−5.02	0.44	93.57
**10**	36.63	10.45	19.78	11.80	3.47	−5.69	−5.48	−5.53	477.82	−0.71	1040.41	−5.11	0.37	95.24

The results of the drug-likeness of the compounds **5**–**10**, including the reference 5RC, computed using SwissADME (49) is given in Table 3. As can be seen, all compounds displayed excellent drug-likeness. None of them violated the parameters required for Ro5/Ro3 (66). Interestingly compound **5**, which also displayed the best activity *in vitro*, showed the lowest topological polar surface area (TPSA = 82.1; limit ≤140 Ǻ^2^), molecular weight (MW = 296.4 amu; limit ≤500) and log*P* (1.38; limit log*P* ≤ 5). Notably, the predicted properties were better for the **5**–**10** than the reference compound 5RC. RADAR plot, in which each vertex shows optimum values of different descriptors, also did not show any deviation (Supplementary Figure S11). Among the main results of QikProp, all compounds show reasonable CNS activity (−1). The average number of HBD and HBA was found to be 3 and 4.5, respectively, which is within the recommended value (67). Solubility profiles like QPlogS (−4.79 to −5.69) and CIQP logS (−4.12 to −5.48) were excellent for all the compounds (recommended value: between −0.5 to −6.5). Similarly, the predicted brain/blood partition coefficient (QPlogBB) and apparent gut-blood barrier permeability QPPCaco (463.10 to 484.04) was high for the studied compounds. The most active compounds **5** and **9** showed low dipole moment and high human oral absorption (%HOA).

Despite having these advantages and drug-like features, we also noted some predicted properties that could undermine their potential. For example, the EGG plot (Supplementary Figure S12) indicates that all compounds are predicted to be well-absorbed by the gastrointestinal tract but have no access to the brain. Besides, PGP is also predicted to pump them out. We computed the toxicity profile of the compounds (Supplementary Table S1). All compounds are predicted to exhibit AMES toxicity, hERG-II toxicity and possible hepato-toxicity (50). QikProp also predicted IC_50_ values for blockage of HERG K^+^ channels. These issues are concerning and will be studied in future investigations.

#### 3.3.4 Density functional theory (DFT) studies

**Figure 10:**
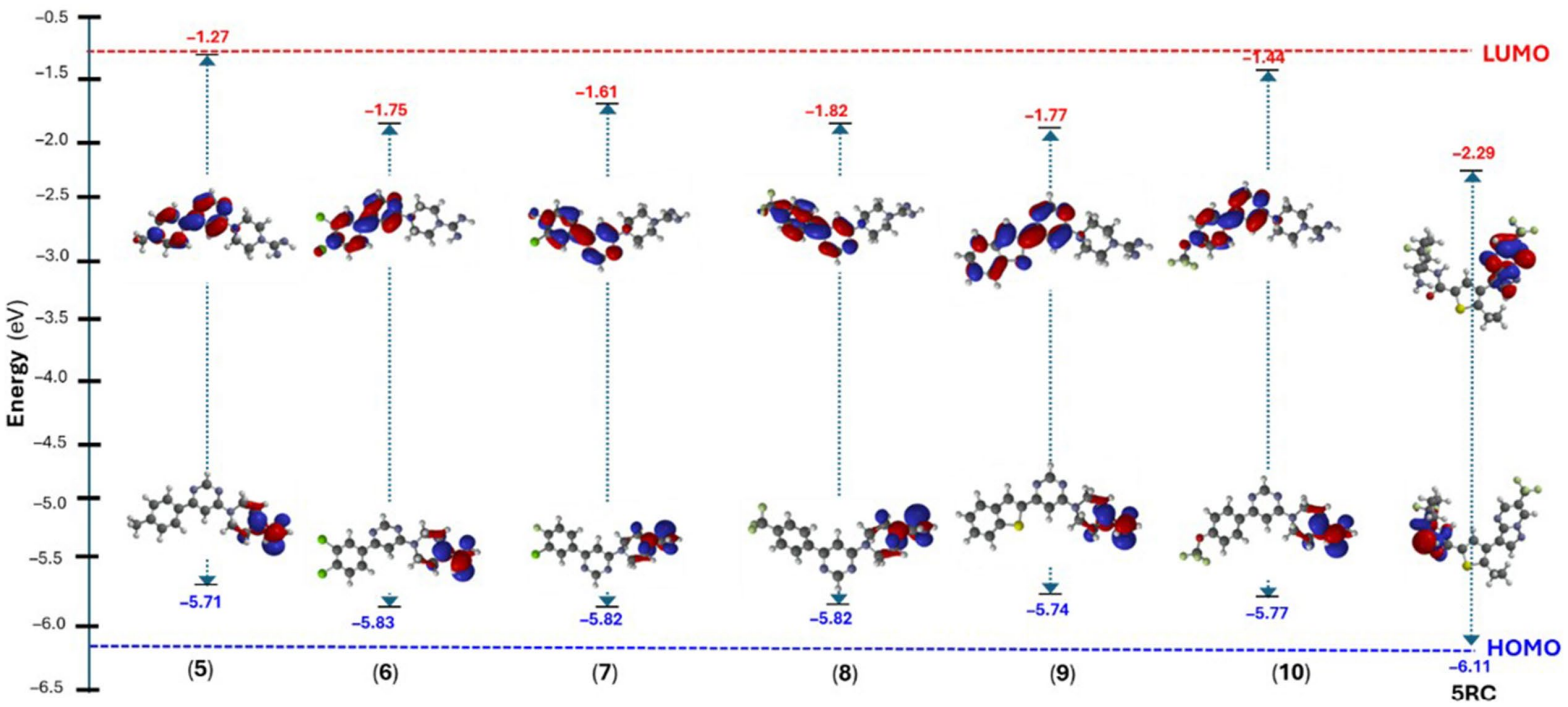
Energy of the frontier molecular orbitals of compound **5**–**10** and 5RC.

Density functional theory (DFT) calculations, like molecular docking and simulation studies, have become routinely used tools in drug discovery programs (68). A plethora of research available shows that DFT can be used in drug modelling (63), designing polymer based drug delivery (69), and calculating numerous other molecular and quantum descriptors (63). In the present study, we computed the energy and distribution of frontiers molecular orbitals, which was then used to estimate the molecular chemical indexes like chemical reactivity, hardness, softness, etc. for the synthesized **5–10** and reference (5RC) compounds. In 5RC, highest occupied molecular orbital (HOMO) was located over 6-amino-2,2-difluorocyclohexyl unit while the lowest unoccupied molecular orbital (LUMO) was over 6-(trifluoromethyl) pyrazolo[1,5-a]pyrimidin-3-yl fragment (Figure 10). The HOMO-LUMO gap, which is a key indicator of a molecule’s chemical stability and reactivity, was found to be 3.82 eV (Supplementary Table S2). In the case of compounds **5**–**10**, both HOMOs/LUMOs were found to increase. Such band gaps have been reported in small molecules recently (70). We can see that the HOMO level of the reference compound (5RC) was deeper, while LUMO was higher than the compounds **5–10**. It is also interesting to note that the most active compounds **5** (−5.71 eV) and **9** (−5.74 eV) have the highest HOMO levels. Among the studied compounds, (**5**) exhibited the highest HOMO-LUMO gap (4.44 eV). Like the reference compound 5RC, here, too, HOMO was located over piperazine-1-carboximidamide while LUMO over 4-(6-(p-tolyl)pyrimidin-4-yl) fragment. Similar observations were noted for the other compounds of series (Supplementary Figures S13, S14). Red regions over the molecules in the electrostatic potential (ESP) map indicate electron-rich region, which participated in the H-bonding, as revealed in the docking studies.

## 4 Conclusion

The design and development of new drug candidates with applications in ADs is a hot area of research. In the present work, we report the synthesis and characterization of pyrimidine-based small molecules as potential inhibitors of MARK4. *In-vitro* ATPase enzyme inhibition assay revealed that the compounds are potent inhibitors of MARK4 with an IC_50_ value in the 5.35 ± 0.22–16.53 ± 1.71 μM range. Fluorescence quenching assay also supported this observation as indicated by the high binding constant (*K* = 1.5 ± 0.51 × 10^5^ M^−1^ for **5** and 1.14 ± 0.26 × 10^5^ M^−1^ for **9**), which is higher than several reported synthetic and natural product-based MARK4 inhibitors. To gain additional insight into the binding of *N*-hetarenes, we conducted a comprehensive *in-silico* studies. Results indicated that compounds are efficient MARK4 binders and stabilize the receptor while maintaining secondary structure elements. Finally, ADME/T was predicted and compared with the experimental bioactivity. We firmly believe that this class of compounds is a promising lead for developing new anti-AD drugs. While this study provides clear evidence that 4,6-disubstitited pyrimidine-based compounds are promising candidates for anti-AD research, further studies (e.g., in-vivo pharmacokinetics) will be required to understand more about the role of functionalities over the molecule. Besides, additional biological/biochemical studies would be needed to shed light on their toxicity and performance in animal models.

## Data Availability

The original contributions presented in the study are included in the article/Supplementary material, further inquiries can be directed to the corresponding author/s.

## References

[1] Gustavsson A Norton N Fast T Frölich L Georges J Holzapfel D. Global estimates on the number of persons across the Alzheimer's disease continuum. Alzheimers Dement. (2023) 19:658–70. doi: 10.1002/alz.12694, PMID: 35652476

[2] Ministry of Health, S.A. (2023). World Alzheimer's Day [Online]. Ministry of Health, Saudi Arabia. Available online at: https://www.moh.gov.sa/en/HealthAwareness/healthDay/2023/Pages/HealthDay-2023-09-21-001.aspx (Accessed October 21, 2024)

[3] Mahoney R Regan C Katona C Livingston G. Anxiety and depression in family caregivers of people with Alzheimer disease: the LASER-AD study. Am J Geriatr Psychiatry. (2005) 13:795–801. doi: 10.1097/00019442-200509000-00008, PMID: 16166409

[4] Reitz C Pericak-Vance MA Foroud T Mayeux R. A global view of the genetic basis of Alzheimer disease. Nat Rev Neurol. (2023) 19:261–77. doi: 10.1038/s41582-023-00789-z, PMID: 37024647 PMC10686263

[5] Alhazmi HA Albratty M. An update on the novel and approved drugs for Alzheimer disease. Saudi Pharmaceut J. (2022) 30:1755–64. doi: 10.1016/j.jsps.2022.10.004, PMID: 36601504 PMC9805975

[6] Zhang J Zhang Y Wang J Xia Y Zhang J Chen L. Recent advances in Alzheimer’s disease: mechanisms, clinical trials and new drug development strategies. Signal Transduct Target Ther. (2024) 9:211. doi: 10.1038/s41392-024-01911-3, PMID: 39174535 PMC11344989

[7] Sarabia-Vallejo Á López-Alvarado P Menéndez JC. Small-molecule theranostics in Alzheimer's disease. Eur J Med Chem. (2023) 255:115382. doi: 10.1016/j.ejmech.2023.115382, PMID: 37141706

[8] Gonçalves PB Sodero ACR Cordeiro Y. Natural products targeting amyloid-β oligomer neurotoxicity in Alzheimer's disease. Eur J Med Chem. (2024) 276:116684. doi: 10.1016/j.ejmech.2024.116684 39032401

[9] Anwar S Shahwan M Hasan GM Islam A Hassan MI. Microtubule-affinity regulating kinase 4: a potential drug target for cancer therapy. Cell Signal. (2022) 99:110434. doi: 10.1016/j.cellsig.2022.110434, PMID: 35961526

[10] Trinczek B Brajenovic M Ebneth A Drewes G. MARK4 is a novel microtubule-associated proteins/microtubule affinity-regulating kinase that binds to the cellular microtubule network and to centrosomes. J Biol Chem. (2004) 279:5915–23. doi: 10.1074/jbc.M304528200, PMID: 14594945

[11] Naz F Anjum F Islam A Ahmad F Hassan MI. Microtubule affinity-regulating kinase 4: structure, function, and regulation. Cell Biochem Biophys. (2013) 67:485–99. doi: 10.1007/s12013-013-9550-7, PMID: 23471664

[12] Oba T Homma D Limlingan SJM Fukuchi A Asada A Saito T. A cell-penetrating peptide derived from SARS-CoV-2 protein Orf9b allosterically inhibits MARK4 activity and mitigates tau toxicity. Neurobiol Dis. (2023) 188:106334. doi: 10.1016/j.nbd.2023.106334, PMID: 37884211

[13] Anwar S Hassan MI Parvez S. Ropinirole reverses the effects of neuroinflammation, and cellular demise by downregulating the MARK4-NFκβ signaling system in Alzheimer's disease. Int J Biol Macromol. (2024) 271:132425 38759860 10.1016/j.ijbiomac.2024.132425

[14] Ahmed S Queen A Irfan I Siddiqui MN Abdulhameed Almuqdadi HT Setia N. Vanillin-Isatin hybrid-induced MARK4 inhibition as a promising therapeutic strategy against hepatocellular carcinoma. ACS Omega. (2024) 9:25945–59. doi: 10.1021/acsomega.4c00661, PMID: 38911744 PMC11190929

[15] Ahmed S Khan P Irfan I Anwar S Shamsi A Arora B. Structure-guided design and development of vanillin-triazole conjugates as potential MARK4 inhibitors targeting hepatocellular carcinoma. J Mol Struct. (2023) 1293:136303. doi: 10.1016/j.molstruc.2023.136303

[16] Aneja B Khan NS Khan P Queen A Hussain A Rehman MT. Design and development of Isatin-triazole hydrazones as potential inhibitors of microtubule affinity-regulating kinase 4 for the therapeutic management of cell proliferation and metastasis. Eur J Med Chem. (2019) 163:840–52. doi: 10.1016/j.ejmech.2018.12.026, PMID: 30579124

[17] Shamsi A Anwar S Mohammad T Alajmi MF Hussain A Rehman MT. MARK4 inhibited by AChE inhibitors, donepezil and Rivastigmine tartrate: insights into Alzheimer’s disease therapy. Biomol Ther. (2020) 10:789. doi: 10.3390/biom10050789, PMID: 32443670 PMC7277793

[18] Alam MZ Bagabir HA Zaher MAF Alqurashi TM Alghamdi BS Ashraf GM. Naringenin as a potent inhibitor molecule for targeting microtubule affinity-regulating kinase 4 (mark4): a molecular docking and in vitro study for therapeutics of Alzheimer's disease. Advance Life Sci. (2024) 11:136–43.

[19] Alrouji M Dasgupta D Ashraf GM Bilgrami AL Alhumaydhi FA Al Abdulmonem W. Inhibition of microtubule affinity regulating kinase 4 by an acetylcholinesterase inhibitor, Huperzine a: computational and experimental approaches. Int J Biol Macromol. (2023) 235:123831. doi: 10.1016/j.ijbiomac.2023.123831, PMID: 36870649

[20] Ashraf GM Dasgupta D Alam MZ Baeesa SS Alghamdi BS Anwar F. Inhibition of microtubule affinity regulating kinase 4 by metformin: exploring the neuroprotective potential of antidiabetic drug through spectroscopic and computational approaches. Molecules. (2022) 27:4652. doi: 10.3390/molecules27144652, PMID: 35889524 PMC9320910

[21] Anwar S Shamsi A Kar RK Queen A Islam A Ahmad F. Structural and biochemical investigation of MARK4 inhibitory potential of cholic acid: towards therapeutic implications in neurodegenerative diseases. Int J Biol Macromol. (2020) 161:596–604. doi: 10.1016/j.ijbiomac.2020.06.078, PMID: 32535203

[22] Anwar S Khan S Shamsi A Anjum F Shafie A Islam A. Structure-based investigation of MARK4 inhibitory potential of Naringenin for therapeutic management of cancer and neurodegenerative diseases. J Cell Biochem. (2021) 122:1445–59. doi: 10.1002/jcb.30022, PMID: 34121218

[23] Shamsi A Dasgupta D Alhumaydhi FA Khan MS Alsagaby SA Al Abdulmonem W. Inhibition of MARK4 by serotonin as an attractive therapeutic approach to combat Alzheimer's disease and neuroinflammation. RSC Med Chem. (2022) 13:737–45. doi: 10.1039/D2MD00053A, PMID: 35814926 PMC9215163

[24] Anwar S Mohammad T Azhar MK Fatima H Alam A Hasan GM. Investigating MARK4 inhibitory potential of Bacopaside II: targeting Alzheimer's disease. Int J Biol Macromol. (2023) 245:125364. doi: 10.1016/j.ijbiomac.2023.125364, PMID: 37315665

[25] Anwar S Shamsi A Shahbaaz M Queen A Khan P Hasan GM. Rosmarinic acid exhibits anticancer effects via MARK4 inhibition. Sci Rep. (2020) 10:10300. doi: 10.1038/s41598-020-65648-z, PMID: 32587267 PMC7316822

[26] Naz F Khan FI Mohammad T Khan P Manzoor S Hasan GM. Investigation of molecular mechanism of recognition between citral and MARK4: a newer therapeutic approach to attenuate cancer cell progression. Int J Biol Macromol. (2018) 107:2580–9. doi: 10.1016/j.ijbiomac.2017.10.143, PMID: 29079437

[27] Khan P Queen A Mohammad T Smita Khan NS Hafeez ZB Hassan MI. Identification of α-mangostin as a potential inhibitor of microtubule affinity regulating kinase 4. J Nat Prod. (2019) 82:2252–61. doi: 10.1021/acs.jnatprod.9b00372, PMID: 31343173

[28] Khan P Rahman S Queen A Manzoor S Naz F Hasan GM. Elucidation of dietary polyphenolics as potential inhibitor of microtubule affinity regulating kinase 4: in silico and in vitro studies. Sci Rep. (2017) 7:9470. doi: 10.1038/s41598-017-09941-4, PMID: 28842631 PMC5573368

[29] Haque A Alenezi KM Rasheed M Abdul SM Rahman MA Anwar S. 4, 6-Disubstituted pyrimidine-based microtubule affinity-regulating kinase 4 (MARK4) inhibitors: synthesis, characterization, in-vitro activity and in-silico studies. Front Pharmacol. (2024) 15:1517504. 39902071 10.3389/fphar.2024.1517504 PMC11788324

[30] Yousuf M Shamsi A Mohammad T Azum N Alfaifi SY Asiri AM. Inhibiting cyclin-dependent kinase 6 by taurine: implications in anticancer therapeutics. ACS Omega. (2022) 7:25844–52. doi: 10.1021/acsomega.2c03479, PMID: 35910117 PMC9330843

[31] Dahiya R Mohammad T Gupta P Haque A Alajmi MF Hussain A. Molecular interaction studies on ellagic acid for its anticancer potential targeting pyruvate dehydrogenase kinase 3. RSC Adv. (2019) 9:23302–15. doi: 10.1039/C9RA02864A, PMID: 35514501 PMC9067284

[32] Chi Z Liu R. Phenotypic characterization of the binding of tetracycline to human serum albumin. Biomacromolecules. (2010) 12:203–9. doi: 10.1021/bm1011568, PMID: 21142141

[33] Sack JS Gao M Kiefer SE Myers JE Newitt JA Wu S. Crystal structure of microtubule affinity-regulating kinase 4 catalytic domain in complex with a pyrazolopyrimidine inhibitor. Acta Crystallographica Section F. (2016) 72:129–34. doi: 10.1107/S2053230X15024747, PMID: 26841763 PMC4741193

[34] Laskowski RA Jabłońska J Pravda L Vařeková RS Thornton JM. PDBsum: structural summaries of PDB entries. Protein Sci. (2018) 27:129–34. doi: 10.1002/pro.3289, PMID: 28875543 PMC5734310

[35] Dundas J Ouyang Z Tseng J Binkowski A Turpaz Y Liang J. CASTp: computed atlas of surface topography of proteins with structural and topographical mapping of functionally annotated residues. Nucleic Acids Res. (2006) 34:W116–8. doi: 10.1093/nar/gkl282, PMID: 16844972 PMC1538779

[36] Morris GM Huey R Lindstrom W Sanner MF Belew RK Goodsell DS. AutoDock4 and AutoDockTools4: automated docking with selective receptor flexibility. J Comput Chem. (2009) 30:2785–91. doi: 10.1002/jcc.21256, PMID: 19399780 PMC2760638

[37] Sanner MF. Python: a programming language for software integration and development. J Mol Graph Model. (1999) 17:57–61. PMID: 10660911

[38] Ahamad S Hassan MI Dwivedi N. Designing of phenol-based β− carbonic anhydrase1 inhibitors through QSAR, molecular docking, and MD simulation approach (2018) 8:1–18. doi: 10.1007/s13205-018-1278-z, PMID: 29765814 PMC5950840

[39] Nguyen NT Nguyen TH Pham TNH Huy NT Bay MV Pham MQ. Autodock vina adopts more accurate binding poses but autodock4 forms better binding affinity. J Chem Inf Model. (2019) 60:204–11. 10.1021/acs.jcim.9b00778 31887035

[40] Delano WL. Pymol: an open-source molecular graphics tool. CCP4 Newsl. Protein Crystallogr. (2002) 40:82–92.

[41] Bowers KJ Chow E Xu H Dror RO Eastwood MP Gregersen BA. Scalable algorithms for molecular dynamics simulations on commodity clusters In: Proceedings of the 2006 ACM/IEEE conference on supercomputing. Tampa, Florida: Association for Computing Machinery (2006).

[42] Schrödinger Release 2024-4. Desmond molecular dynamics system, D.E.S.R., New York, Ny, 2024. Schrödinger, New York, NY,: Maestro-Desmond Interoperability Tools (2024).

[43] Glättli A Daura X Van Gunsteren WF. Derivation of an improved simple point charge model for liquid water: SPC/a and SPC/L. J Chem Phys. (2002) 116:9811–28. doi: 10.1063/1.1476316

[44] Harder E Damm W Maple J Wu C Reboul M Xiang JY. OPLS3: a force field providing broad coverage of drug-like small molecules and proteins. J Chem Theory Comput. (2016) 12:281–96. doi: 10.1021/acs.jctc.5b00864, PMID: 26584231

[45] Lu C Wu C Ghoreishi D Chen W Wang L Damm W. OPLS4: improving force field accuracy on challenging regimes of chemical space. J Chem Theory Comput. (2021) 17:4291–300. doi: 10.1021/acs.jctc.1c00302, PMID: 34096718

[46] Hoover WG. Canonical dynamics: equilibrium phase-space distributions. Phys Rev A. (1985) 31:1695–7. doi: 10.1103/PhysRevA.31.1695, PMID: 9895674

[47] Martyna GJ Tobias DJ Klein ML. Constant pressure molecular dynamics algorithms. J Chem Phys. (1994) 101:4177–89. doi: 10.1063/1.467468

[48] Nosé S. A unified formulation of the constant temperature molecular dynamics methods. J Chem Phys. (1984) 81:511–9. doi: 10.1063/1.447334

[49] Daina A Michielin O Zoete V. SwissADME: a free web tool to evaluate pharmacokinetics, drug-likeness and medicinal chemistry friendliness of small molecules. Sci Rep. (2017) 7:1–13. doi: 10.1038/srep42717, PMID: 28256516 PMC5335600

[50] Pires DE Blundell TL Ascher DB. pkCSM: predicting small-molecule pharmacokinetic and toxicity properties using graph-based signatures. J Med Chem. (2015) 58:4066–72. doi: 10.1021/acs.jmedchem.5b00104, PMID: 25860834 PMC4434528

[51] Omoboyowa DA. Exploring molecular docking with E-pharmacophore and QSAR models to predict potent inhibitors of 14-α-demethylase protease from *Moringa* spp. Pharmacol Res Modern Chin Med. (2022) 4:100147. doi: 10.1016/j.prmcm.2022.100147

[52] Shahbazi S Kaur J Kuanar A Kar D Singh S Sobti RC. Risk of late-onset Alzheimer's disease by plasma cholesterol: rational in silico drug investigation of pyrrole-based HMG-CoA reductase inhibitors. Assay Drug Dev Technol. (2017) 15:342–51. doi: 10.1089/adt.2017.804, PMID: 29077483

[53] Hehre WJ Huang WW. Chemistry with computation: An introduction to SPARTAN Wavefunction Incorporated. Irvine, CA, USA (1995).

[54] Ayala-Aguilera CC Valero T Lorente-Macias A Baillache DJ Croke S Unciti-Broceta A. Small molecule kinase inhibitor drugs (1995–2021): medical indication, pharmacology, and synthesis. J Med Chem. (2021) 65:1047–131. doi: 10.1021/acs.jmedchem.1c00963, PMID: 34624192

[55] Gong Z Xie Z Qiu J Wang G. Synthesis, biological evaluation and molecular docking study of 2-substituted-4, 6-diarylpyrimidines as α-glucosidase inhibitors. Molecules. (2017) 22:1865. doi: 10.3390/molecules22111865, PMID: 29084182 PMC6150375

[56] Pant S Kapri A Nain S. Pyrimidine analogues for the management of neurodegenerative diseases. Eur J Med Chem Rep. (2022) 6:100095. doi: 10.1016/j.ejmcr.2022.100095

[57] Kumar J Gill A Shaikh M Singh A Shandilya A Jameel E. Pyrimidine-Triazolopyrimidine and pyrimidine-pyridine hybrids as potential acetylcholinesterase inhibitors for Alzheimer's disease. ChemistrySelect. (2018) 3:736–47. doi: 10.1002/slct.201702599

[58] BoläNder A Kieser D Voss C Bauer S SchöN C Burgold S. Bis (arylvinyl) pyrazines,-pyrimidines, and-pyridazines as imaging agents for tau fibrils and β-amyloid plaques in Alzheimer’s disease models. J Med Chem. (2012) 55:9170–80. doi: 10.1021/jm300653b, PMID: 22913544

[59] Devi B Jangid K Kumar V Arora T Kumar N Dwivedi AR. Phenylstyrylpyrimidine derivatives as potential multipotent therapeutics for Alzheimer's disease. RSC Med Chem. (2024) 15:2922–36. doi: 10.1039/D4MD00277F, PMID: 39149109 PMC11324047

[60] Kumar B Kumar V Prashar V Saini S Dwivedi AR Bajaj B. Dipropargyl substituted diphenylpyrimidines as dual inhibitors of monoamine oxidase and acetylcholinesterase. Eur J Med Chem. (2019) 177:221–34. doi: 10.1016/j.ejmech.2019.05.039, PMID: 31151057

[61] Albani JR. Structure and dynamics of macromolecules: Absorption and fluorescence studies. Amsterdam, The Netherlands: Elsevier (2011).

[62] Jenardhanan P Mannu J Mathur PP. The structural analysis of MARK4 and the exploration of specific inhibitors for the MARK family: a computational approach to obstruct the role of MARK4 in prostate cancer progression. Mol BioSyst. (2014) 10:1845–68. doi: 10.1039/C3MB70591A, PMID: 24763618

[63] Arias F Franco-Montalban F Romero M Carrión MD Camacho ME. Synthesis, bioevaluation and docking studies of new imidamide derivatives as nitric oxide synthase inhibitors. Bioorg Med Chem. (2021) 44:116294. doi: 10.1016/j.bmc.2021.116294, PMID: 34218000

[64] Roncaglioni A Toropov AA Toropova AP Benfenati E. In silico methods to predict drug toxicity. Curr Opin Pharmacol. (2013) 13:802–6. doi: 10.1016/j.coph.2013.06.001 23797035

[65] Tian S Wang J Li Y Li D Xu L Hou T. The application of in silico drug-likeness predictions in pharmaceutical research. Adv Drug Deliv Rev. (2015) 86:2–10. doi: 10.1016/j.addr.2015.01.009, PMID: 25666163

[66] Lipinski CA Lombardo F Dominy BW Feeney PJ. Experimental and computational approaches to estimate solubility and permeability in drug discovery and development settings. Adv Drug Deliv Rev. (2012) 64:4–17. doi: 10.1016/j.addr.2012.09.019 11259830

[67] Shahbazi S Kaur J Singh S Achary KG Wani S Jema S. Impact of novel N-aryl piperamide NO donors on NF-κB translocation in neuroinflammation: rational drug-designing synthesis and biological evaluation. Innate Immun. (2018) 24:24–39. doi: 10.1177/1753425917740727, PMID: 29145791 PMC6830765

[68] Sabe VT Ntombela T Jhamba LA Maguire GE Govender T Naicker T. Current trends in computer aided drug design and a highlight of drugs discovered via computational techniques: a review. Eur J Med Chem. (2021) 224:113705. doi: 10.1016/j.ejmech.2021.113705, PMID: 34303871

[69] Adekoya OC Adekoya GJ Sadiku ER Hamam Y Ray SS. Application of DFT calculations in designing polymer-based drug delivery systems: an overview. Pharmaceutics. (2022) 14:1972. doi: 10.3390/pharmaceutics14091972, PMID: 36145719 PMC9505803

[70] Nadeem S Anwar A Khan MU Hassan AU Alrashidi KA. Synergistic charge-transfer dynamics of novel pyridoquinazolindone-containing triphenylamine-based push–pull chromophores: from structural optimization to performance metrics in photovoltaic solar cells and static, dynamic, solvent-dependent nonlinear optical response applications. RSC Adv. (2024) 14:32482–500. doi: 10.1039/D4RA05290K 39403151 PMC11472850

