# Supplementary material for: Experimental and theoretical studies on structural changes in the microtubule affinity-regulating kinase 4 (MARK4) protein induced by N-hetarenes: a new class of therapeutic candidates for Alzheimer’s disease
Source: Front Med (Lausanne). 2025 Mar 19;12:1529845. doi: 10.3389/fmed.2025.1529845 (PMC11962044; doi:10.3389/fmed.2025.1529845)
Supplement: Supplementary file 1 [file Data_Sheet_1.PDF]

Experimental and theoretical studies on structural changes in the microtubule affinity-regulating kinase 4 (MARK4) protein induced by *N*-hetarenes: A new class of therapeutic candidates for Alzheimer's disease

Ashanul Haque,[1,*] Khalaf M. Alenezi,[1] Mohd. Saeed Maulana Abdul Rasheed[2], Md. Ataur Rahman[3], Saleha Anwar[4], Shahzaib Ahamad,[5] Dinesh Gupta[5]

[1] Department of Chemistry, College of Science, University of Hail, Kingdom of Saudi Arabia.

[2] Department of Biology, College of Science, University of Hail, Kingdom of Saudi Arabia.

[3] Chemistry Program, New York University Abu Dhabi (NYUAD), Saadiyat Island, United Arab Emirates.

[4] Centre for Interdisciplinary Research in Basic Sciences, New Delhi, India

[5] Translational Bioinformatics Group, International Centre for Genetic Engineering and Biotechnology (ICGEB), Aruna Asaf Ali Marg 110067 New Delhi, India

Supporting information

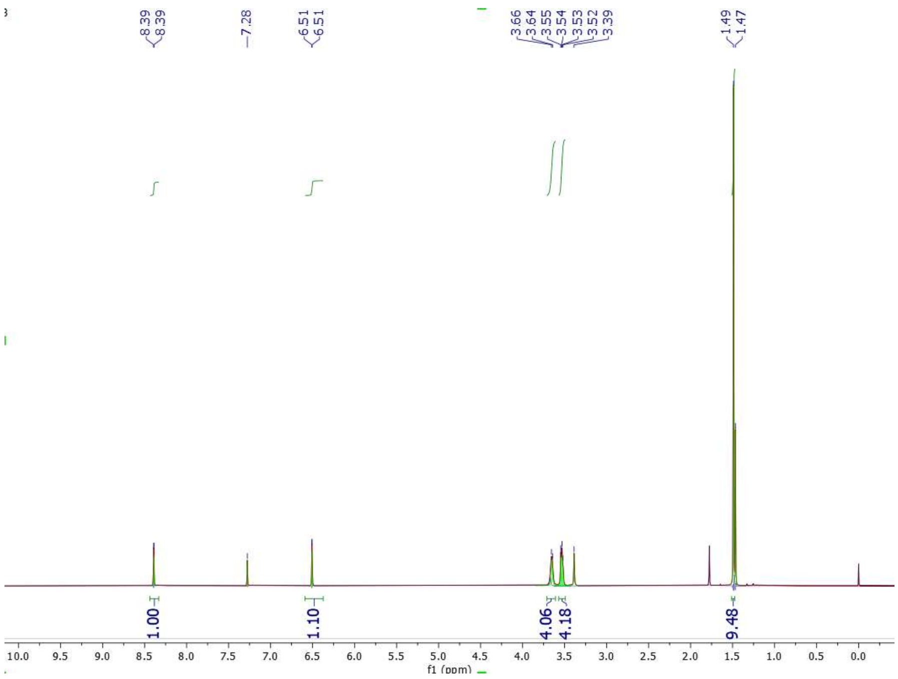

(a)

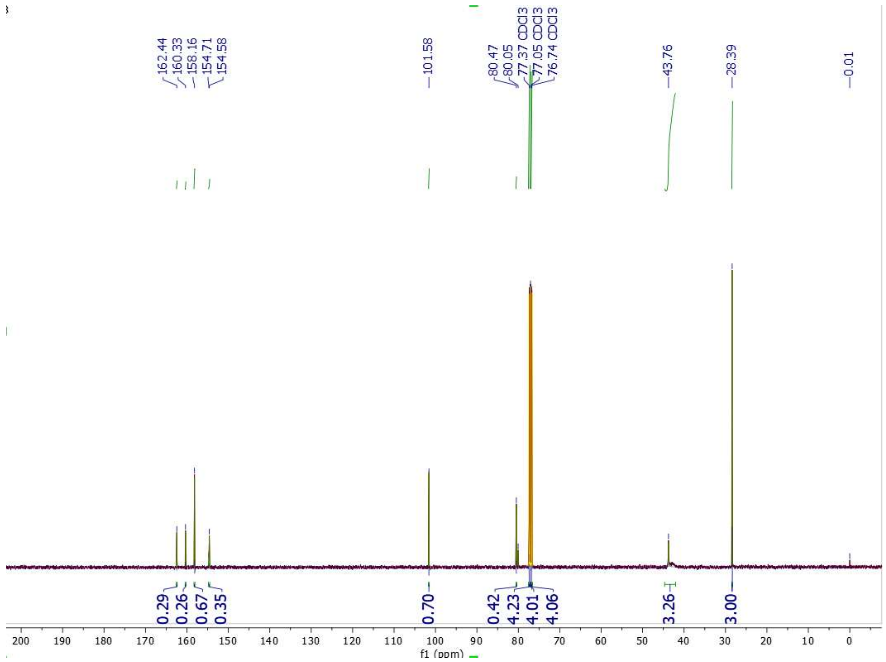

(b)

Figure SF1: (a) ^{1}H-NMR and (b) ^{13}C-NMR of compound **1** collected in $CDCl_3$.

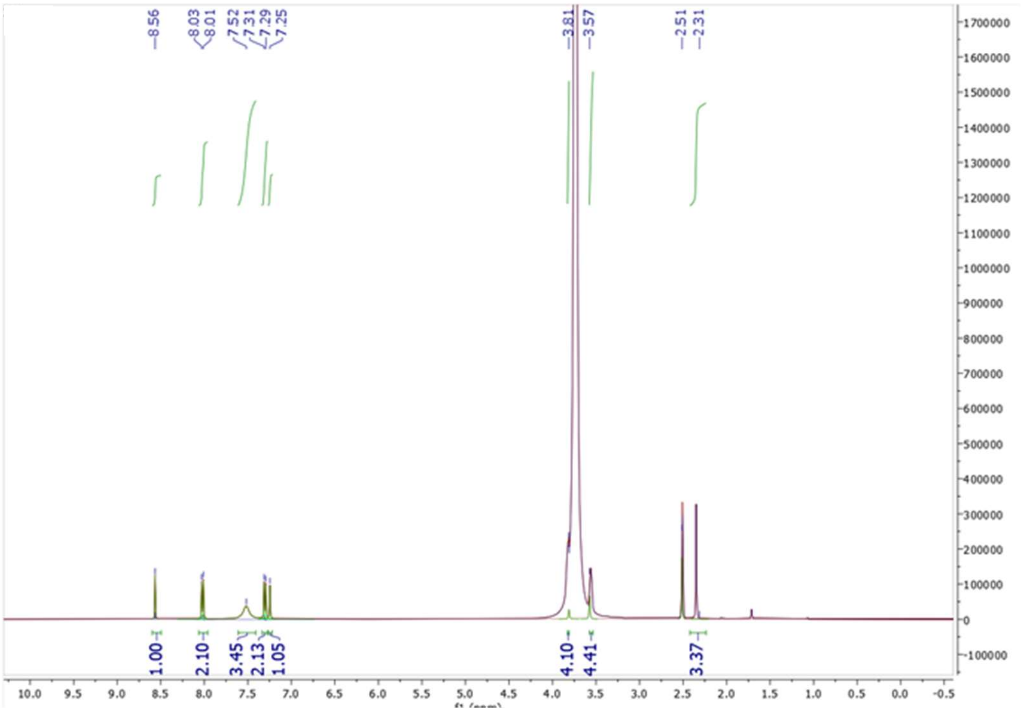

(a)

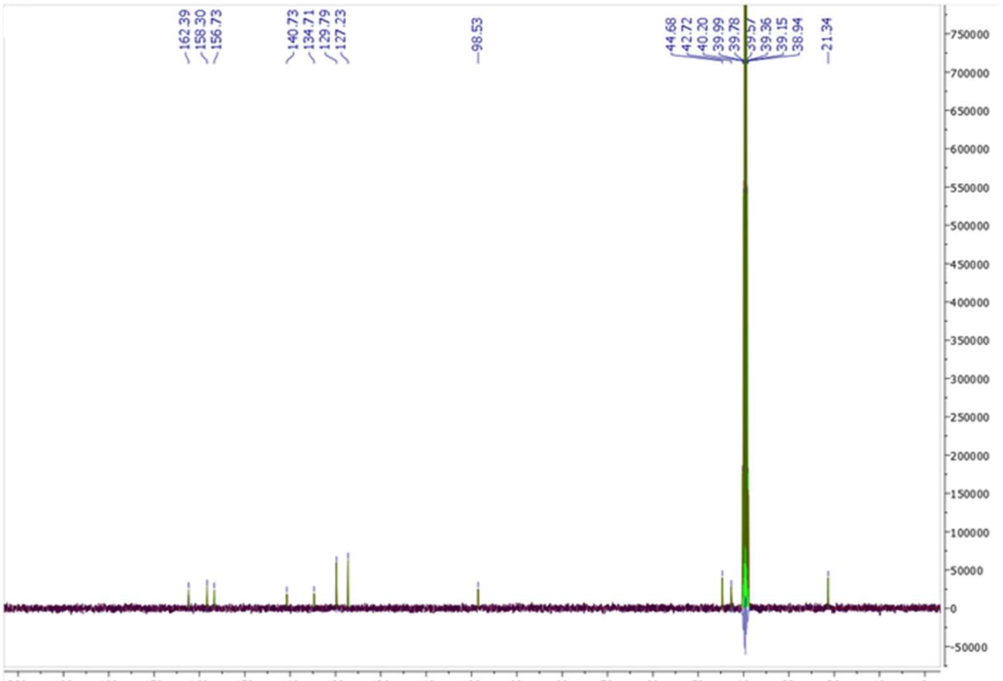

(b)

Figure SF2: (a) ^{1}H-NMR and (b) ^{13}C-NMR of compound **5** collected in DMSO-d_6.

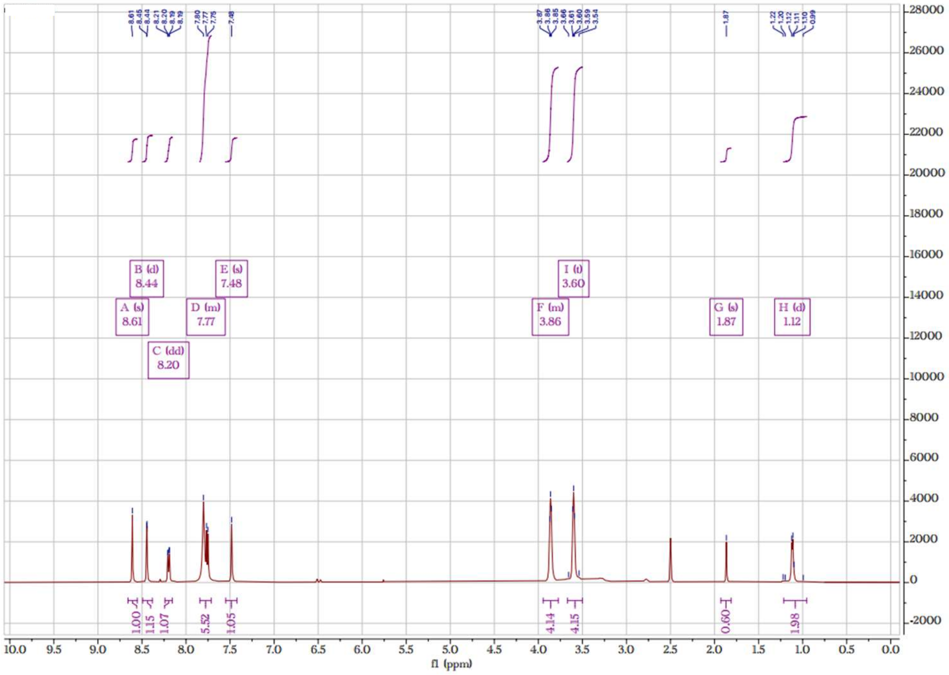

(a)

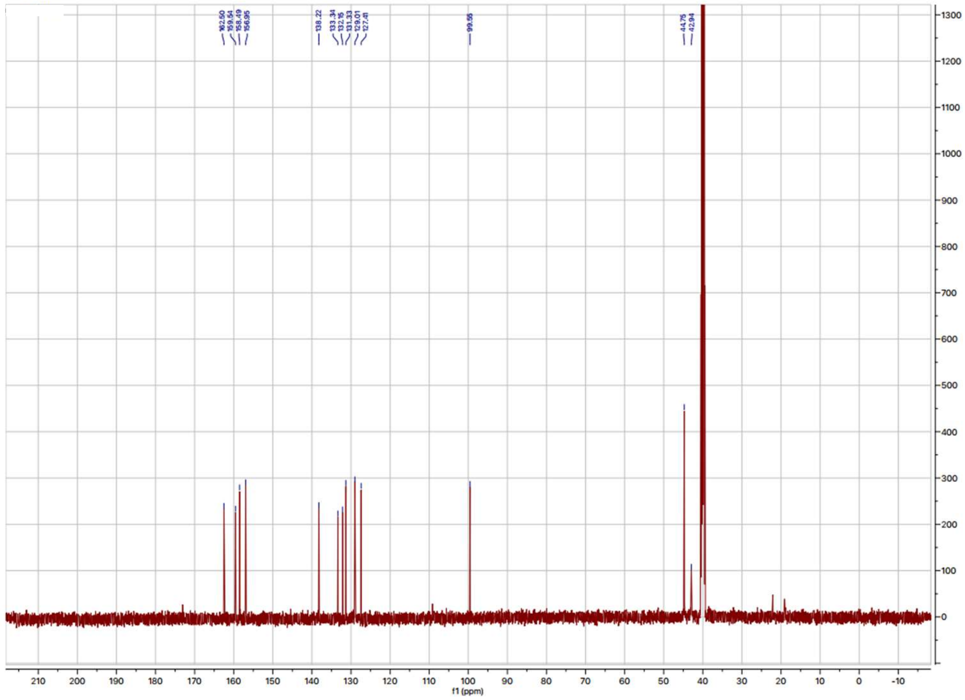

(b)

Figure SF3: (a) ^{1}H-NMR and (b) ^{13}C-NMR of compound **6** collected in DMSO-d_6.

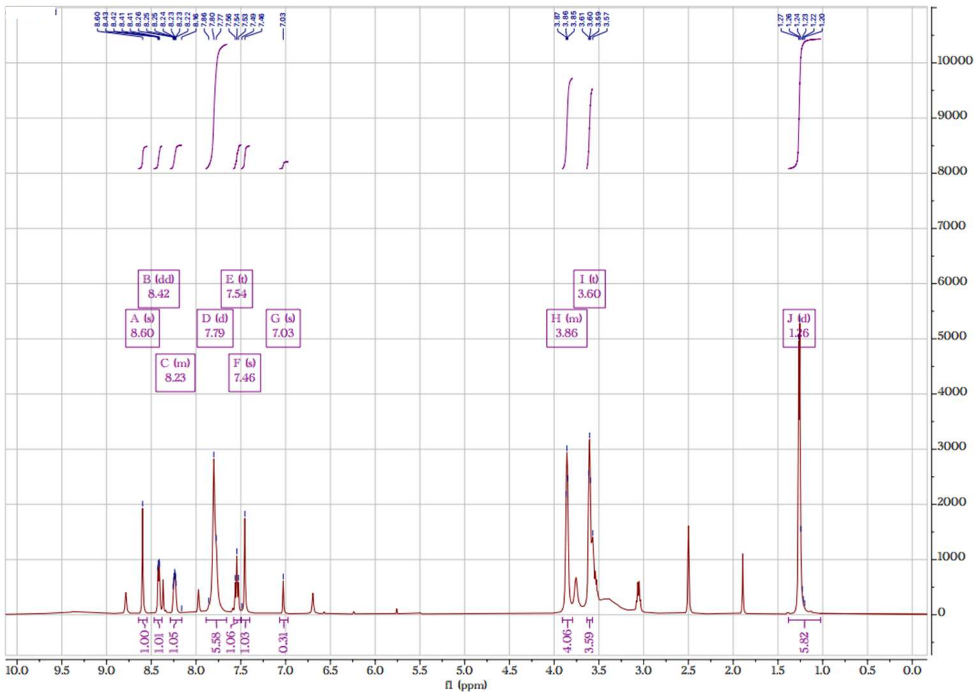

(a)

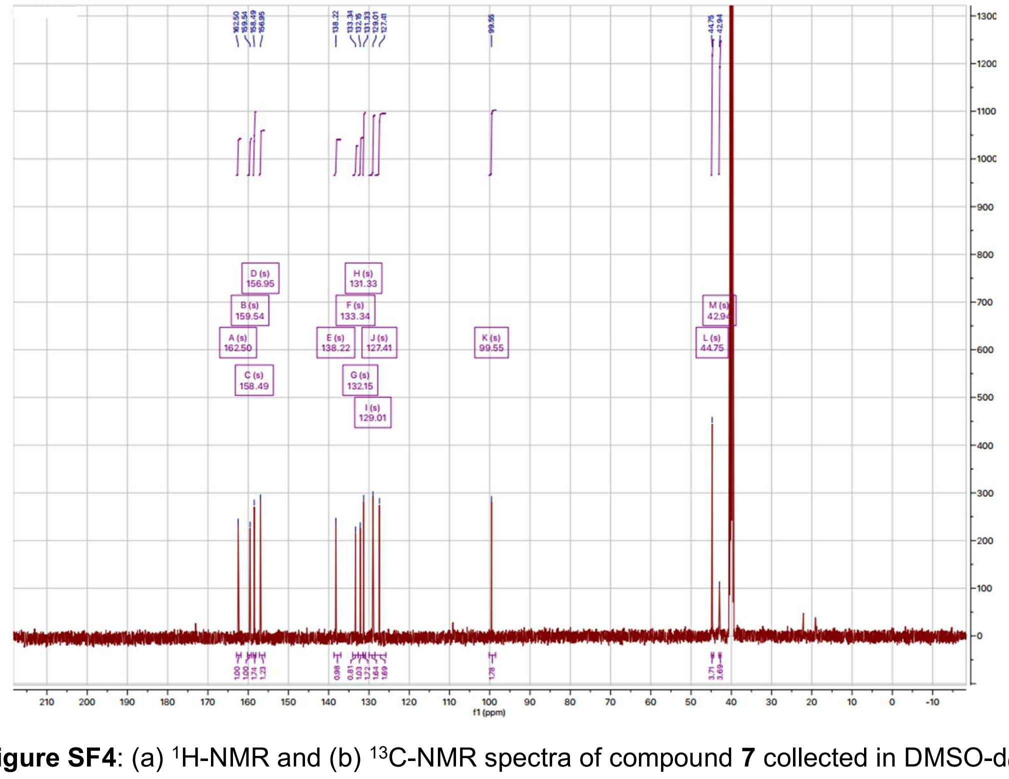

Figure SF4: (a) ^{1}H-NMR and (b) ^{13}C-NMR spectra of compound **7** collected in DMSO-d_6.

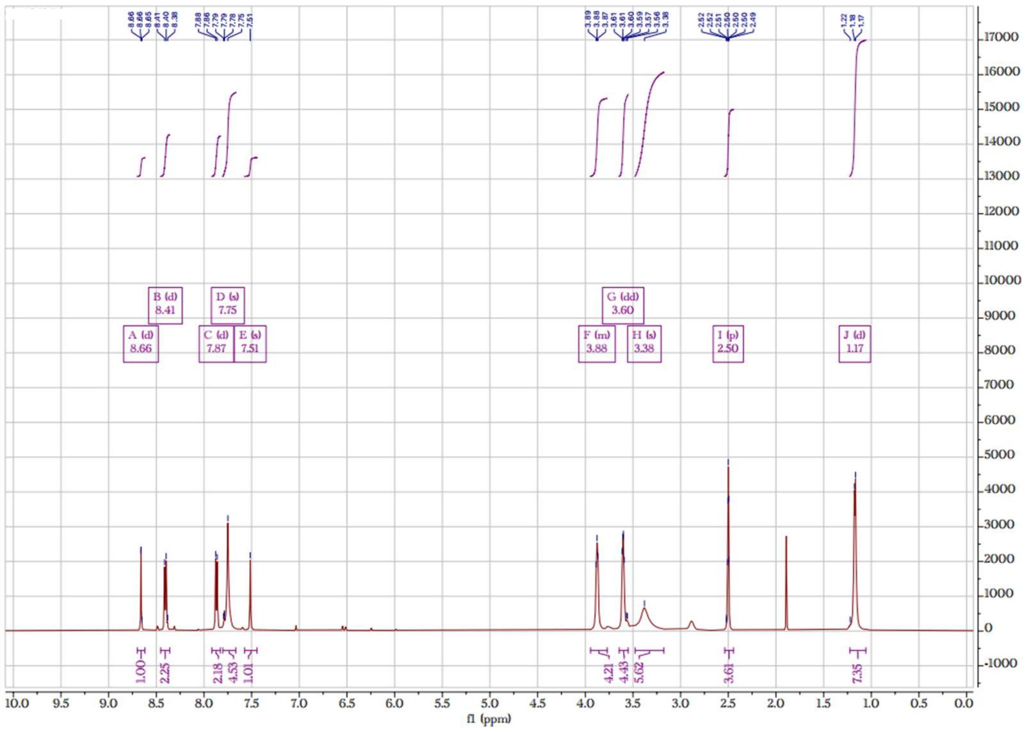

(a)

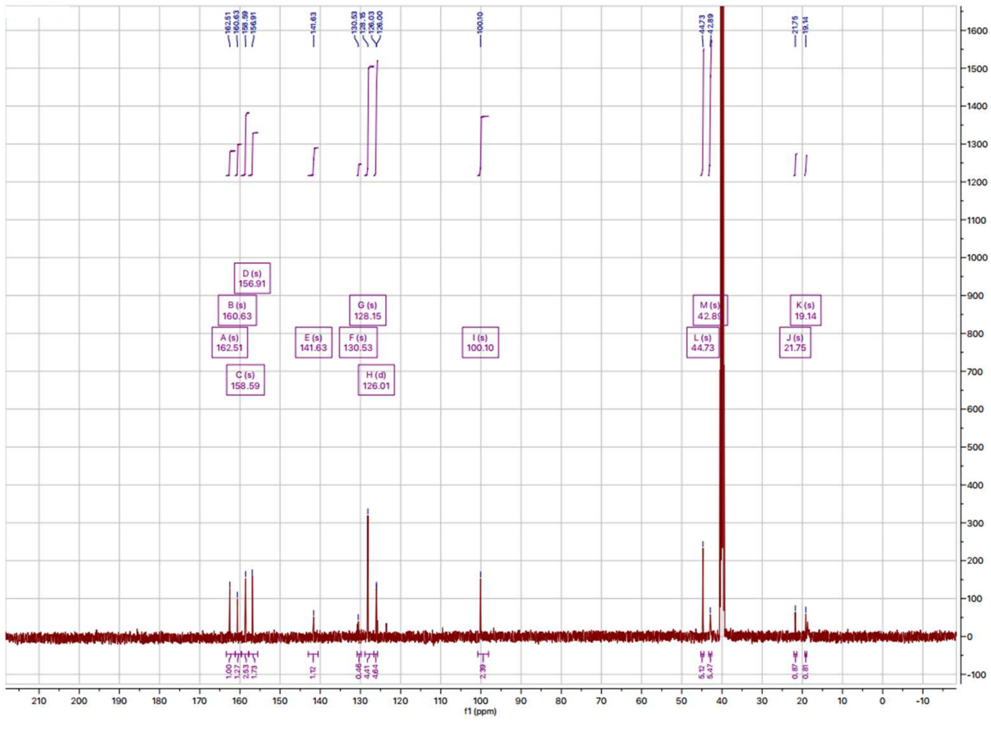

(b)

Figure SF5: (a) ^{1}H-NMR and (b) ^{13}C-NMR spectra of compound **8** collected in DMSO-d_6.

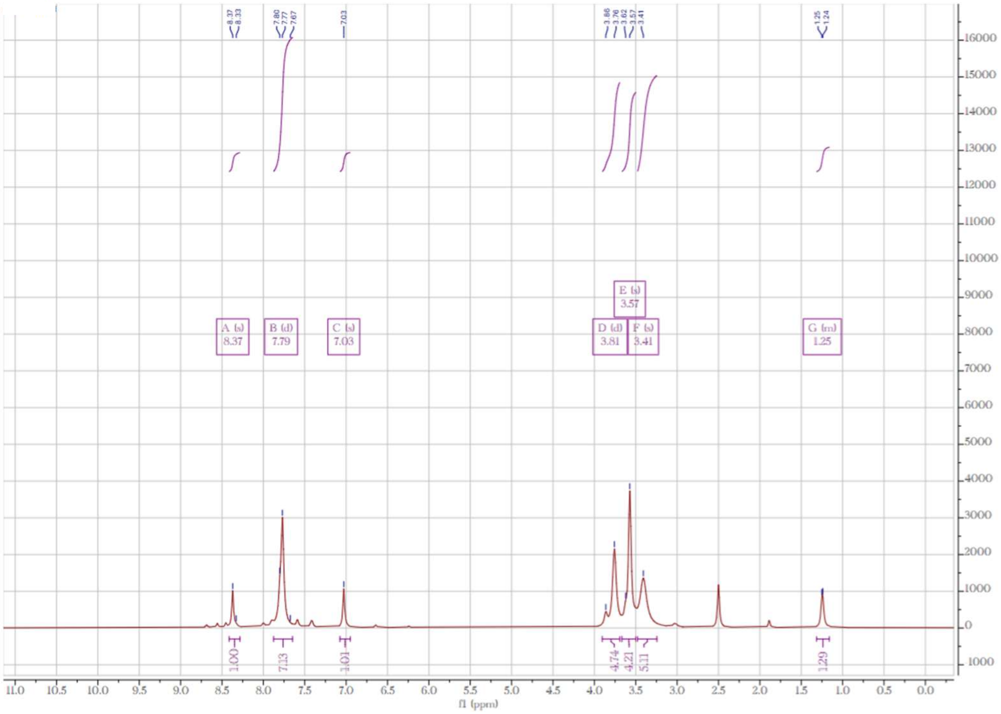

(a)

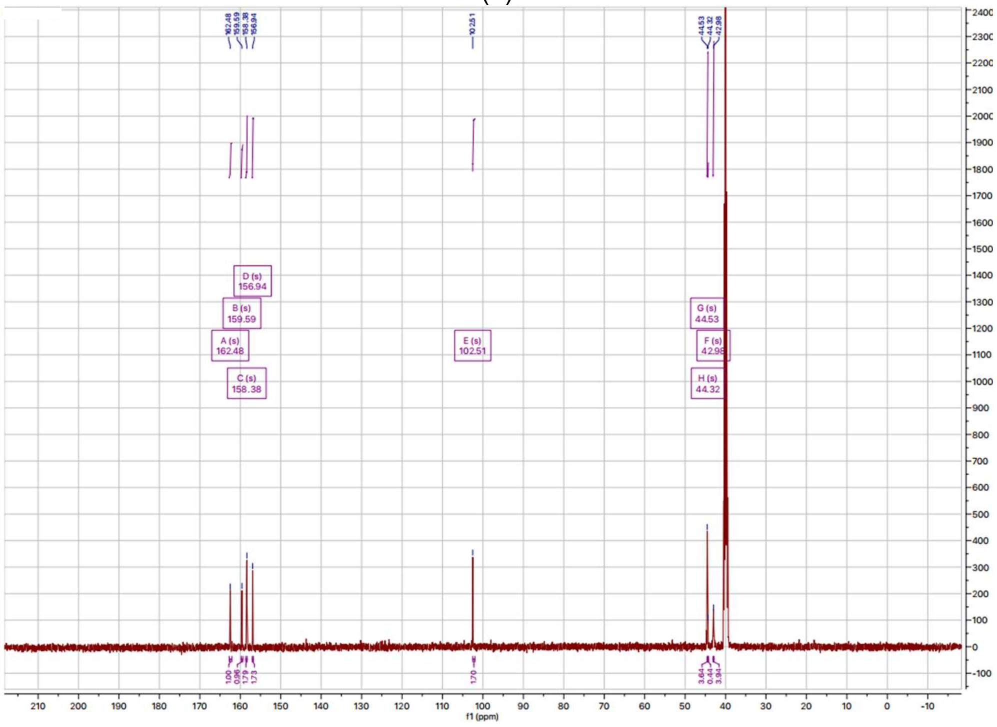

(b)

Figure SF6: (a) ^{1}H-NMR and (b) ^{13}C NMR spectra compound **9** collected in DMSO-d_6.

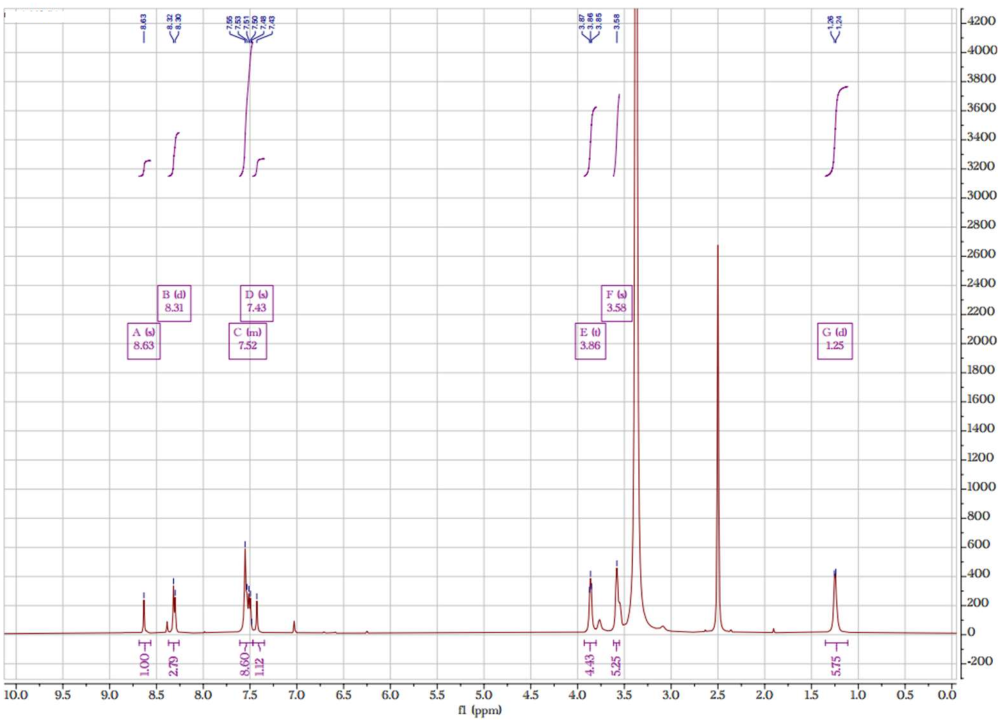

(a)

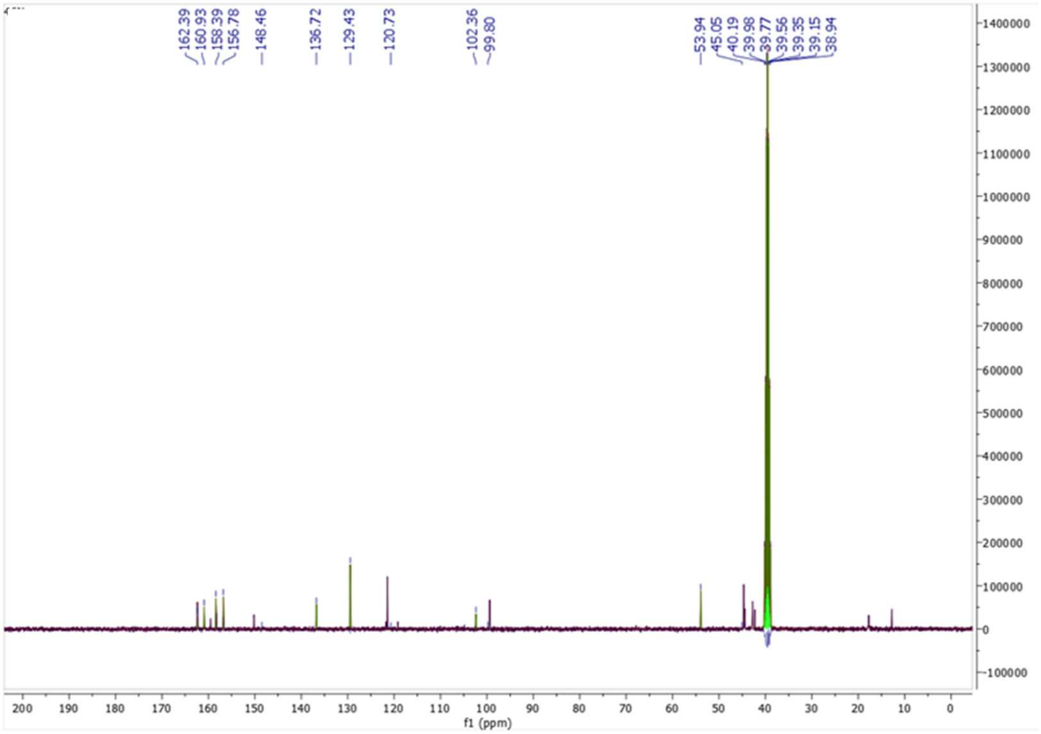

(b)

Figure SF7: (a) ^{1}H-NMR and (b) ^{13}C-NMR spectra compound **10** collected in DMSO-d_6.

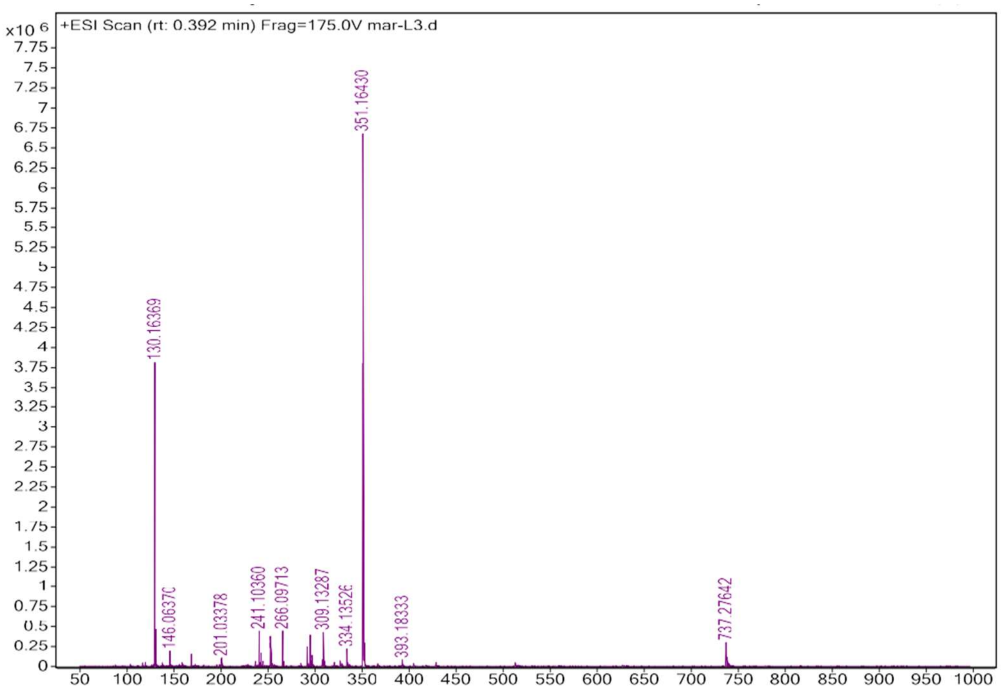
+ESI Scan (rt: 0.392 min) Frag=175.0V mar-L3.d
x10 6
130.16369
146.0637C
201.03378
241.10360
266.09713
309.13287
334.1352€
351.16430
393.18333
737.27642

(a)

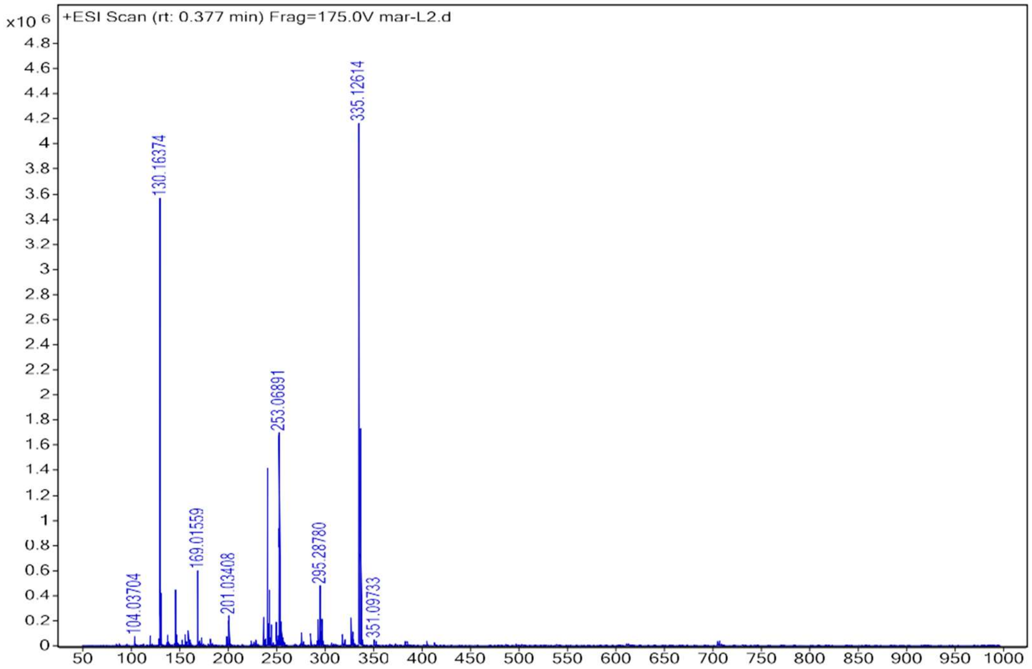
+ESI Scan (rt: 0.377 min) Frag=175.0V mar-L2.d
x10 6
104.03704
130.16374
169.01559
201.03408
253.06891
295.28780
335.12614
351.09733

(b)

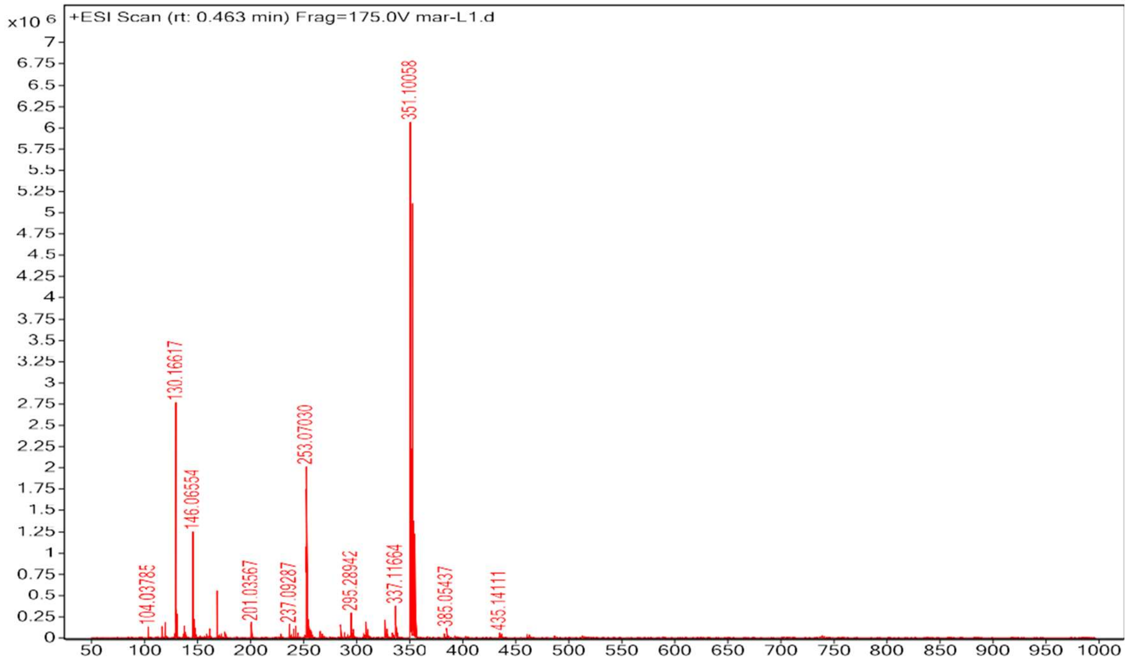

+ESI Scan (rt: 0.463 min) Frag=175.0V mar-L1.d
x10 6
351.10058
130.16617
253.07030
146.06554
104.03785
201.03567
237.09287
295.28942
337.11664
385.05437
435.14111

(c)

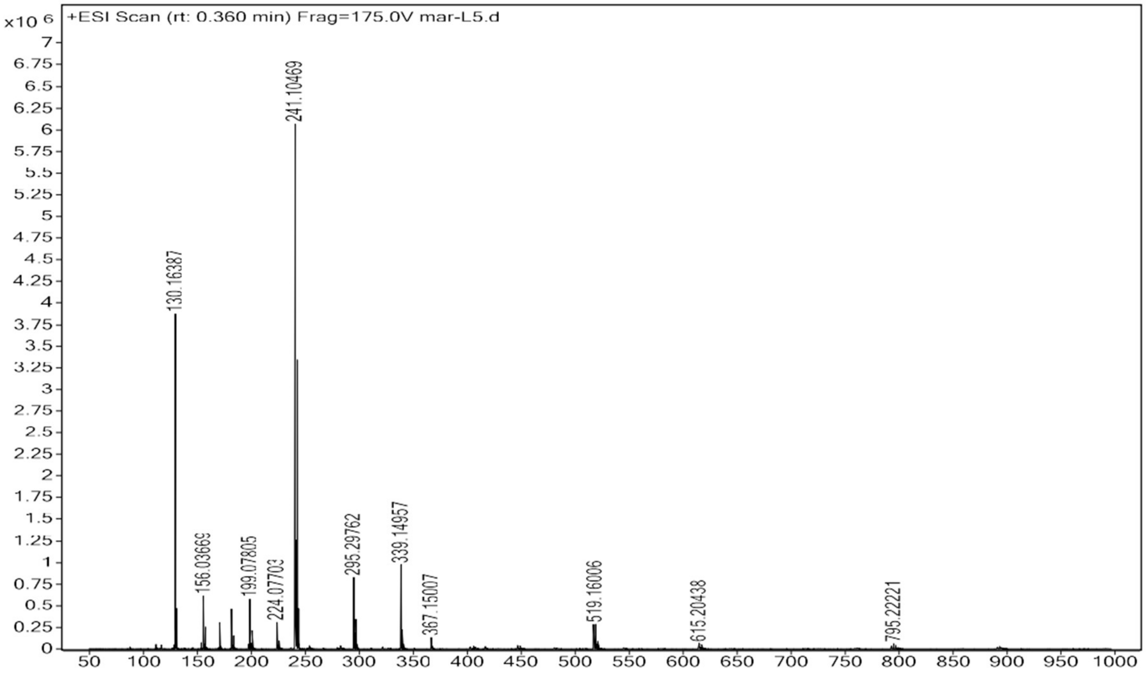

+ESI Scan (rt: 0.360 min) Frag=175.0V mar-L5.d
x10 6
241.10469
130.16387
156.03669
199.07805
224.07703
295.29762
339.14957
367.15007
519.16006
615.20438
795.22221

(d)

.

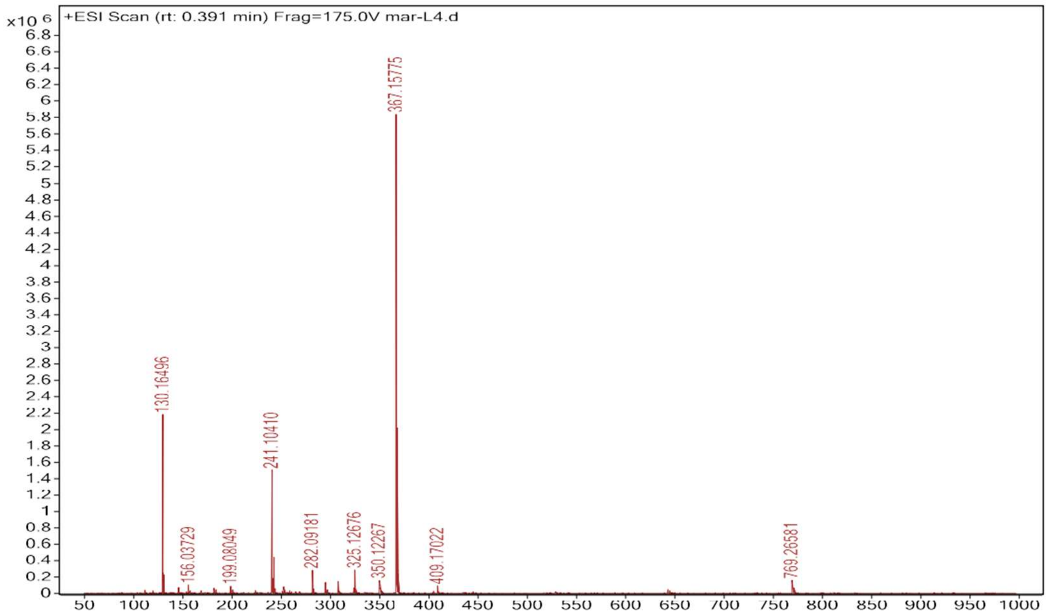

(e)

Figure SF8: Mass spectra of 6-10.

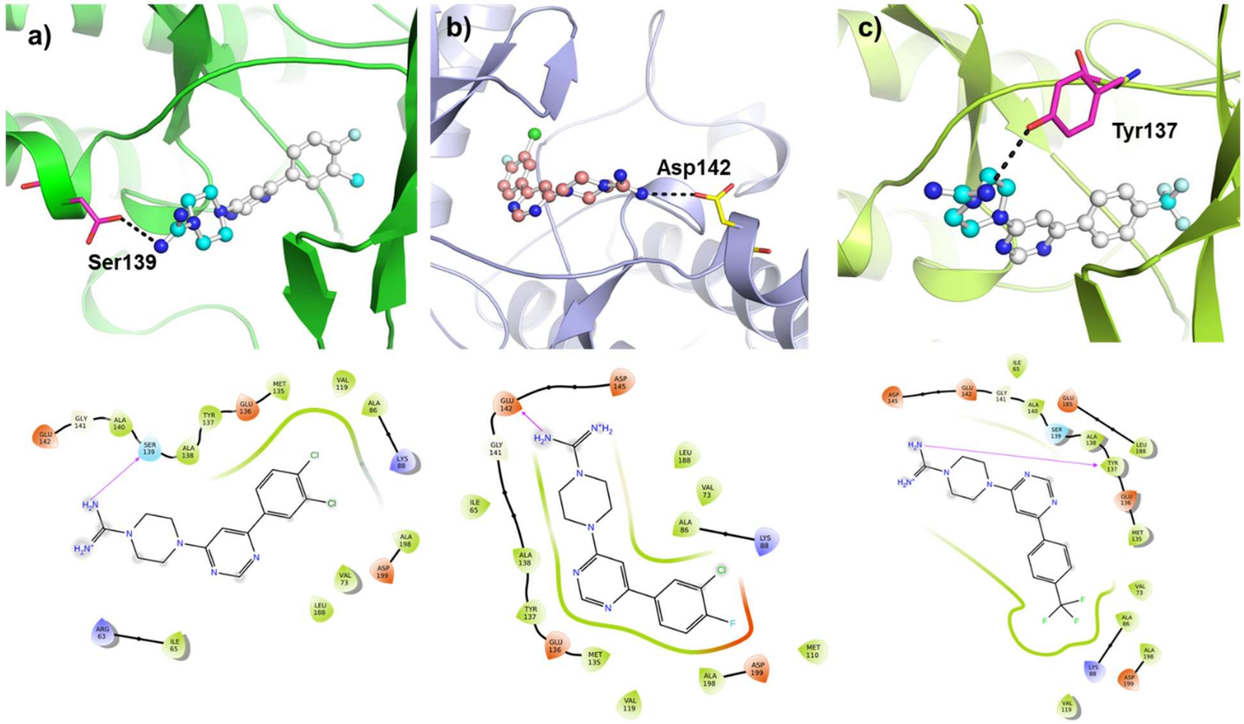

Figure SF9: Interaction between MARK4 (PDB: 5ES1) with compounds 6, 7, and 8.

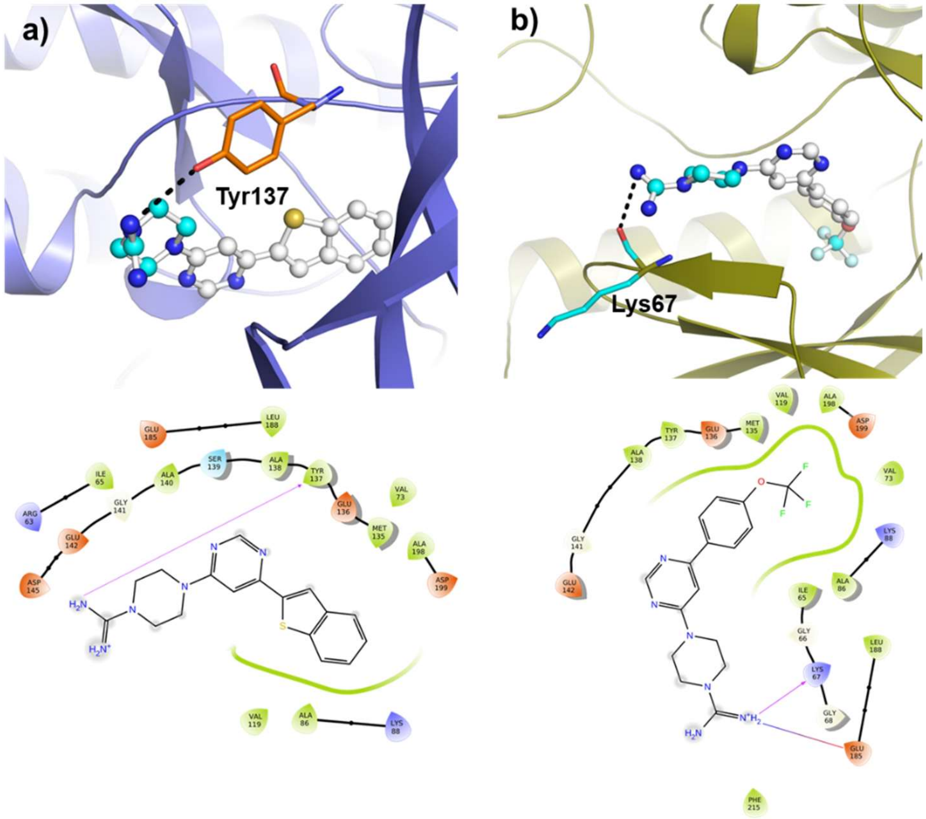

Figure SF10: Interaction between MARK4 with compounds 9, and 10.

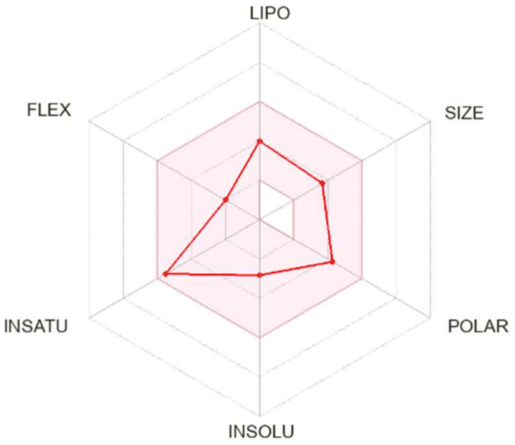
LIPO
SIZE
POLAR
INSOLU
INSATU
FLEX
(**5**)

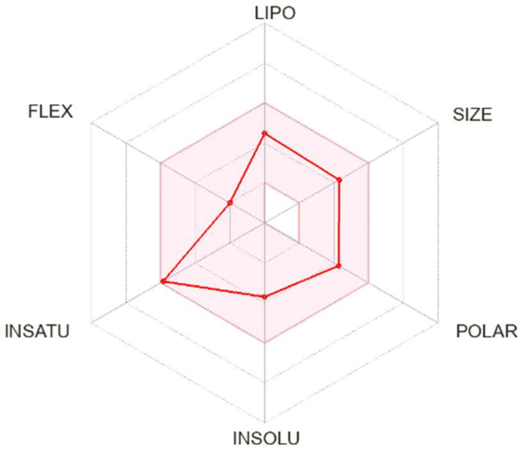
LIPO
SIZE
POLAR
INSOLU
INSATU
FLEX
(**6**)

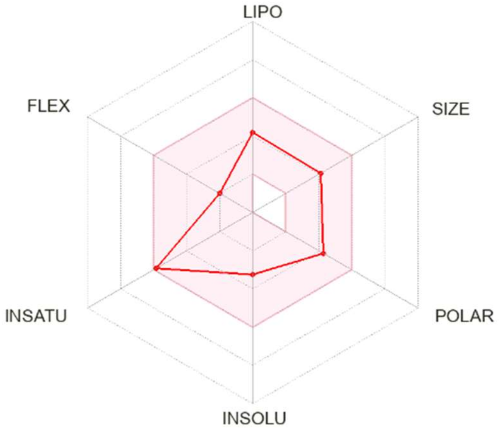
LIPO
SIZE
POLAR
INSOLU
INSATU
FLEX
(**7**)

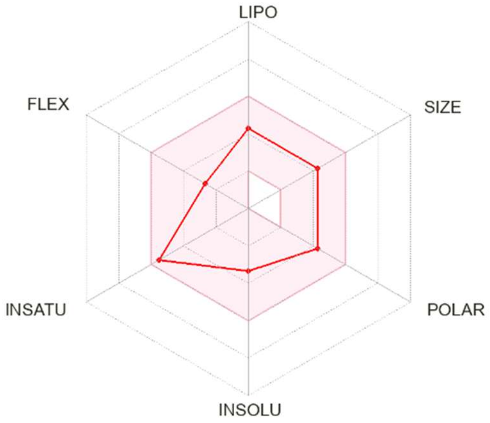
LIPO
SIZE
POLAR
INSOLU
INSATU
FLEX
(**8**)

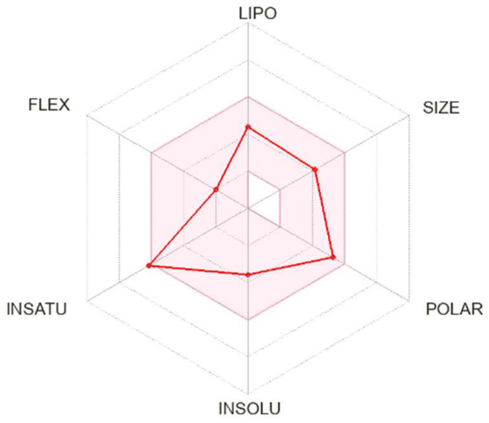
LIPO
SIZE
POLAR
INSOLU
INSATU
FLEX
(**9**)

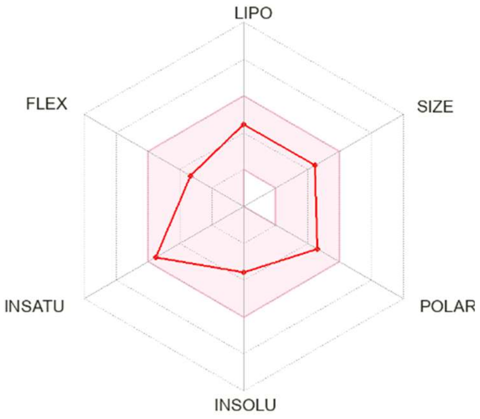
LIPO
SIZE
POLAR
INSOLU
INSATU
FLEX
(**10**)

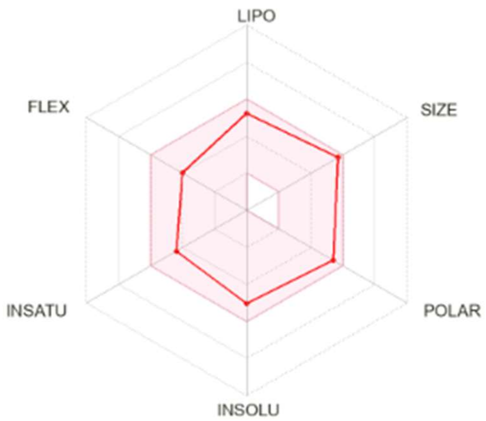

(**5RC**)

Figure SF11: RADAR plots of compounds 5-10 and 5RC.

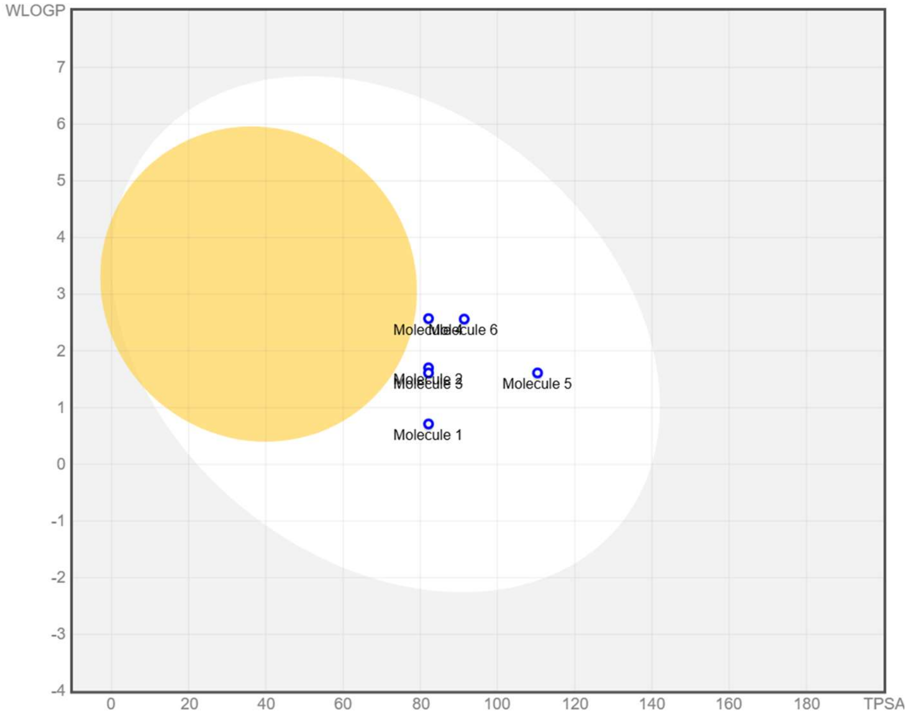

(**5-10**)

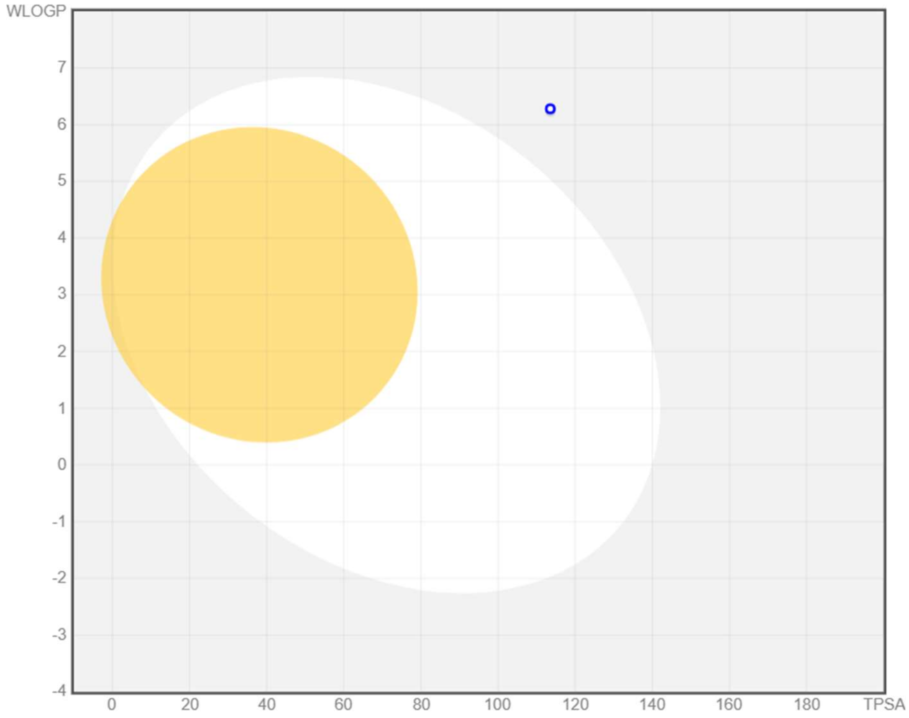

(**5RC**)

Figure SF12: BOILED-EGG diagram of compounds 5-10 and 5RC.

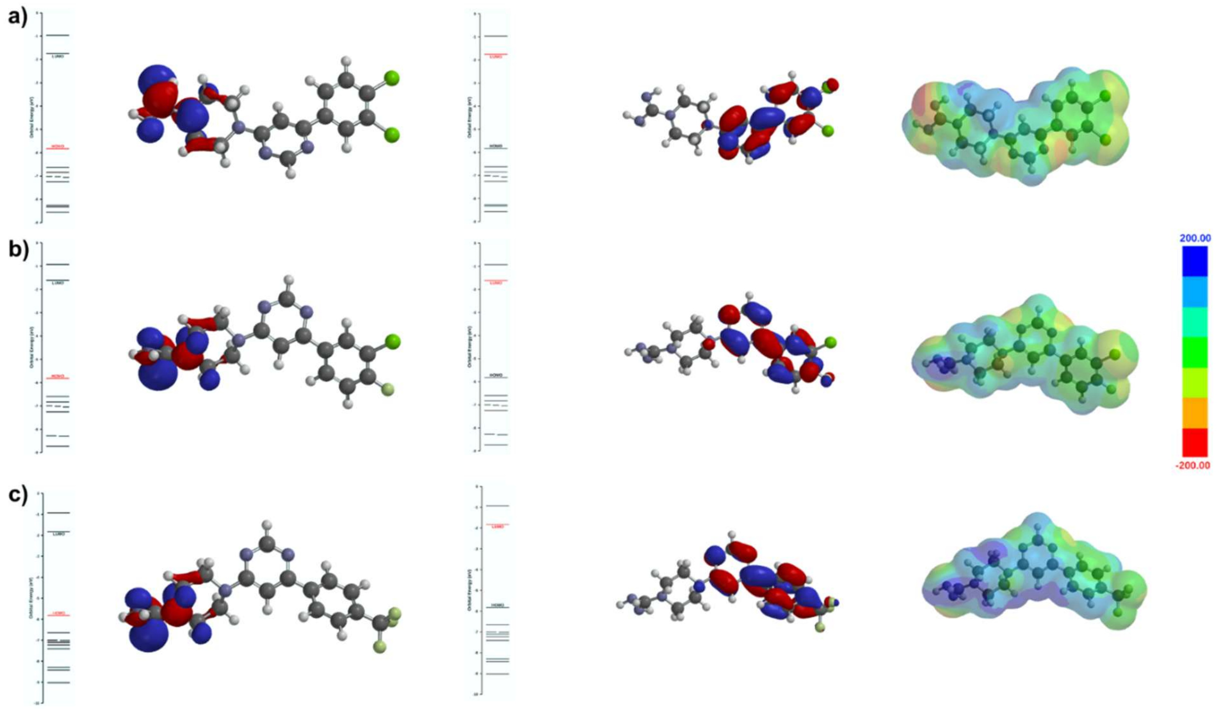

Figure S13: Frontier orbitals, their energy, and ESP of compounds 6, 7, 8 (a-c, respectively).

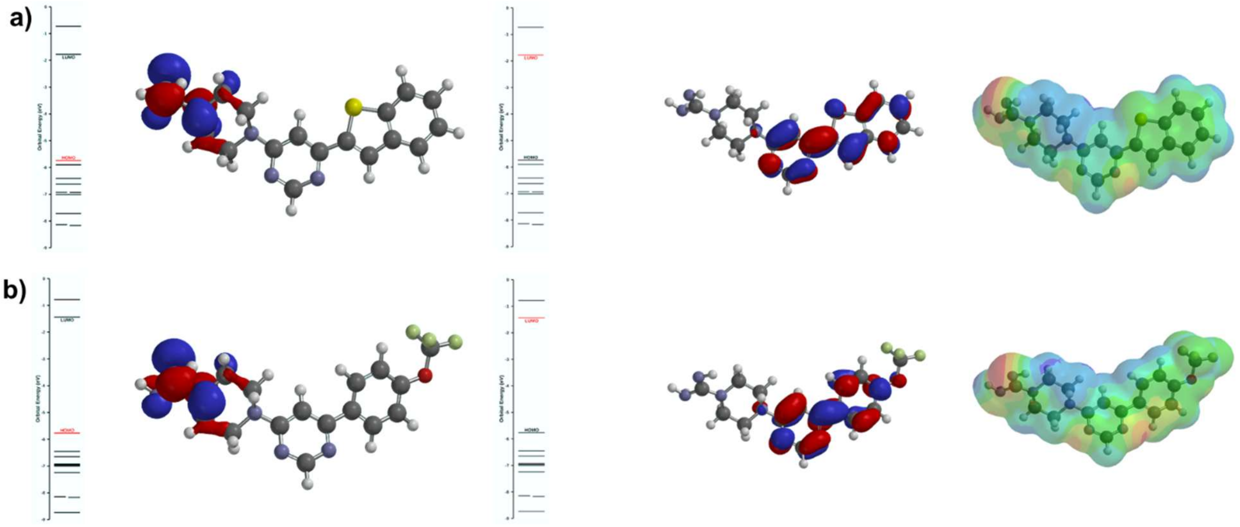

Figure SF14: Frontier orbitals, their energy, and ESP of compounds 9, 10 (a-b, respectively).

Table ST1: Toxicity prediction of the reference and screened compounds using pKCSM.(Pires et al., 2015)

Code #	Parameters[a]									
	AT	MTD	hERG	hERG II	ORAT	CORAT	Hep	SS	TPT	MT
**5**	Yes	-0.274	No	Yes	2.712	2.98	Yes	No	0.285	2.477
**6**	Yes	-0.278	No	Yes	2.788	2.959	Yes	No	0.285	1.782
**7**	Yes	-0.247	No	Yes	2.765	3.04	Yes	No	0.285	2.115
**8**	Yes	-0.322	No	Yes	2.519	0.888	Yes	No	0.593	3.809
**9**	No	-0.221	No	Yes	2.283	1.02	Yes	No	0.49	4.98
**10**	Yes	-0.231	No	Yes	2.489	0.806	Yes	No	0.476	3.812

[a]For abbreviations and details, see ref.(Alshammari et al., 2021)

Table ST2. Chemical reactivity parameters were calculated by density functional theory (DFT)/B3LYP (Becke's three-parameter hybrid exchange functional with the Lee–Yang–Parr correlation functional) method with a polarisation basis set 6-31G* in the gas phase.[a]

Code #	E_{HOMO} (eV)	E_{LUMO} (eV)	ΔE (eV)	I (eV)	A (eV)	X (eV)	η (eV)	σ (eV^{-1})	μ (eV)	ω (eV)
**5**	–5.71	–1.27	4.44	5.71	1.27	3.49	2.22	0.23	-3.49	2.74
**6**	–5.83	–1.75	4.08	5.83	1.75	3.79	2.04	0.25	-3.79	3.52
**7**	–5.82	–1.61	4.21	5.82	1.61	3.71	2.11	0.24	-3.71	3.26
**8**	–5.82	–1.82	4.00	5.82	1.82	3.82	2.00	0.25	-3.82	3.64
**9**	–5.74	–1.77	3.97	5.74	1.77	3.75	1.99	0.25	-3.75	3.54
**10**	–5.77	–1.44	4.33	5.77	1.44	3.60	2.17	0.23	-3.60	2.99
**5RC**	–6.11	-2.29	3.82	6.11	2.29	4.20	1.91	0.26	-4.20	4.61

[a]For abbreviations and details, see ref.(Alshammari et al., 2021)

References

Alshammari, M.M., Soury, R., Alenezi, K.M., Mushtque, M., Rizvi, M.M.A., and Haque, A. (2021). Synthesis, characterization, anticancer and in silico studies of a pyrazole-tethered thiazolidine-2,4-dione derivative. *J Biomol Struct Dyn*, 1-8.

Pires, D.E., Blundell, T.L., and Ascher, D.B. (2015). pkCSM: predicting small-molecule pharmacokinetic and toxicity properties using graph-based signatures. *Journal of Medicinal Chemistry* 58, 4066-4072.
